# A Minimal Set of Tissue-Specific Hypomethylated CpGs Constitute Epigenetic Signatures of Developmental Programming

**DOI:** 10.1371/journal.pone.0072670

**Published:** 2013-09-12

**Authors:** Alejandro Colaneri, Tianyuan Wang, Vijayakanth Pagadala, Jaya Kittur, Nickolas G. Staffa, Shyamal D. Peddada, Elvira Isganaitis, Mary Elizabeth Patti, Lutz Birnbaumer

**Affiliations:** 1 Laboratory of Neurobiology, National Institute of Environmental Health Sciences, Research Triangle Park, North Carolina, United States of America; 2 Biostatistics Branch, National Institute of Environmental Health Sciences, Research Triangle Park, North Carolina, United States of America; 3 Joslin Diabetes Center, Harvard Medical School, Boston, Massachusetts, United States of America; Université Paris-Diderot, France

## Abstract

**Background:**

Cell specific states of the chromatin are programmed during mammalian development. Dynamic DNA methylation across the developing embryo guides a program of repression, switching off genes in most cell types. Thus, the majority of the **t**issue **s**pecific **d**ifferentially **m**ethylated **s**ites (TS-DMS) must be un-methylated CpGs.

**Methodology and Principal Findings:**

Comparison of **e**xpanded **M**ethyl **S**ensitive **C**ut **C**ounting data (eMSCC) among four tissues (liver, testes, brain and kidney) from three C57BL/6J mice, identified 138,052 differentially methylated sites of which 23,270 contain CpGs un-methylated in only one tissue (TS-DMS). Most of these CpGs were located in intergenic regions, outside of promoters, CpG islands or their shores, and up to 20% of them overlapped reported active enhancers. Indeed, tissue-specific enhancers were up to 30 fold enriched in TS-DMS. Testis showed the highest number of TS-DMS, but paradoxically their associated genes do not appear to be specific to the germ cell functions, but rather are involved in organism development. In the other tissues the differentially methylated genes are associated with tissue-specific physiological or anatomical functions. The identified sets of TS-DMS quantify epigenetic distances between tissues, generated during development. We applied this concept to measure the extent of reprogramming in the liver of mice exposed to *in utero* or early postnatal nutritional stress. Different protocols of food restriction reprogrammed the liver methylome in different but reproducible ways.

**Conclusion and Significance:**

Thus, each identified set of differentially methylated sites constituted an epigenetic signature that traced the developmental programing or the early nutritional reprogramming of each exposed mouse. We propose that our approach has the potential to outline a number of disease-associated epigenetic states. The composition of differentially methylated CpGs may vary with each situation, behaving as a composite variable, which can be used as a pre-symptomatic marker for disease.

## Introduction

Animals develop from repeated division of a single cell (a fertilized egg) as a result of precise spatio-temporal regulation of gene expression. Combinatorial use of *cis* and *trans* DNA regulatory elements permit commitment of cells to different lineages. Once a differentiation path is chosen the trajectory of gene expression is maintained through many cell generations. The acquisition of this “cellular memory” is key for the assembly of the different organized tissues, and is maintained largely by epigenetic marks such as 5-methyl cytosine methylation [1]–[3]. During development the genome is faithfully replicated millions of times but the epi-genome varies with each cell type. Moreover, in contrast to the genome, the epigenome is dynamic and sensitive to the environment. Exposure to stress during critical stages of development could cause subtle changes in the epigenome resulting in a small but time-cumulative effect on cellular physiology [1], [2], [4]–[6]. Thus, small deviations in tissue-specific methylation patterns could contribute to the developmental origins of many adult diseases [7], [8].

To understand the role of DNA methylation in the cell-type-specification of the chromatin or in the onset of pathophysiological mechanism, it is necessary to contrast the distribution of methyl-marks across various tissues and different conditions. The ultimate goal will be to identify tissue-specific regulatory loci controlled by methylation. Most of the research done to address this problem has focused on the function of promoters and their CpG islands (CGIs), shaping the perception that the CGIs are the hotspots for epigenetic regulation of tissue specific transcriptional activity [9] [10]–[14]. However, technological advances in methylome analysis have increasingly shown Tissue Specific Differentially Methylated Regions (TS-DMR) as hypo-methylated loci existing outside promoters and CGIs, [15]–[18]. We recently showed that most of the hypo-methylated loci existing in the liver of an adult mouse are located in introns or intergenic regions and do not meet commonly accepted definitions of CGIs [15]. Indeed these CpG-poor un-methylated loci showed the highest concentrations in DNA regulatory sequences, many of which were liver specific [15]. We hypothesized that the epigenetic regulation of transcriptional networks that specify tissues occurs primarily in CpG located outside promoters.

Comparing the distribution of methylation sites in mouse gDNA derived from liver, kidney, brain and testis we found 138,052 independent differentially methylated sites (DMS). One sixth of them consisted of CpGs whose methylation levels were particularly high or low in only one tissue, hence named tissue specific differentially methylated sites (TS-DMS). Most of these are un-methylated CpGs associated with tissue-specific expressed genes. Functional profiles obtained from these genes suggest that relevant aspects of tissue physiology would be epigenetically demarcated during development. The distribution of methylation among a minimal set of 23,270 CpG showed commonalities between biological replicates and differences between tissues, suggesting that these TS-DMS constitute unique epigenetic footprints created during development. We detected further reprogramming of methylation patterns drove by *in utero* and/or immediate postnatal food restriction. We suggest that our approach can outline a number of disease-associated epigenetic states. The composition of DMS would be different for each situation representing highly specific DNA methylation biomarker panels. Discovery of novel epigenetic makers is an area of increasing interest in biomedical research, however most of the newly discovered candidates have been found by focusing on a small number of well-defined loci, usually promoters and CGIs [19]. As these loci can be identified either as methylated or un-methylated, they offer a limited sensitivity or specificity for diagnosis, [20], [21]. On the other hand, a composite variable, such as the composition of CpGs in a set of differentially methylated sites, can represent a larger number of different situations covering wider applications.

## Materials and Methods

### Samples

Four tissues: brain (B), kidney (K), liver (L) and testis (T) were obtained from each of three adult male C57Bl/6J mice. Fetal livers were also derived from the same mouse strain. In addition, 16 liver samples were used from three-week-old male ICR mice (Harlan, Indianapolis, IN, USA) that were subjected to a study of nutritional influences on diabetes and obesity risk [22], [23]. This study was carried out in strict accordance with the recommendations in the Guide for the Care and Use of Laboratory Animals of the National Institutes of Health. Animal protocols “Proposal # 2009-0017 LN, DNA methylation in obesity and diabetes”, were approved by The National Institute of Environmental Health Sciences Animal Care and Use Committee.

### Genome-wide DNA methylation profiling using eMSCC

CpG tag libraries for eMSCC were prepared as described [15], including the addition of equimolar amounts of un-methylated lambda phage DNA (Promega, Cat.# D1521). All libraries were sequenced by Expression Analysis (Durham, NC, USA) using an Illumina Genome Analyzer IIx. Reads (30 to 50 million per library) were aligned to the mouse reference genome (mm9) using MOM [24].

### Identification of TS-DMS

This procedure has four main steps: **1)** For each of 12 samples (3 per tissue), we normalized the reads at each CpG by a factor that is proportional to the total number of reads in the sample; **2)** We aligned all the reads in each sample to Takai-Jones CGIs (TJ-CGIs) and generated the distribution of the average normalized methyl-sensitive-counts (digestion frequencies) of TJ-CGIs. We chose the value at the valley of the distribution as the cutoff to filter out CpGs presumed heavily methylated in all four tissues; **3)** For each CpG that passed the filter in step 2, we performed all six pairwise comparisons of the four tissues, i.e. B-K, B-L, B-T, K-L, K-T, L-T; where B, K, T and L represent brain, kidney, testis and liver respectively and B-K, B-L, etc., represent the pair wise comparisons. For each CpG, the average and the standard deviation of the reads were calculated using all samples. The standardized residual for each sample was then calculated by dividing the difference between the observed numbers of reads for that sample and the corresponding average by the standard deviation. We plotted the quantiles of the residuals against the quantiles of standard normal distribution to see if it is reasonable to assume that the data are approximately normally distributed; **4)** We applied the mdFDR method to identify DMSs based on the p values of all six possible t-tests. The mdFDR procedure allowed us to control the overall false discovery rate for all pairwise comparisons as well as the directional errors when declaring more or less methylated states [25]. In this manner, we identified the preliminary DMS. We next removed the pairs of comparison with average normalized read differences between two tissues that were less than the cutoff value generated from step 2, to ensure that two tissues had a different methylation state at that CpG. Last, for each DMS we averaged reads of each tissue and identified the sites with methylation levels significantly high or low in only one tissue. These sites were called TS-DMS.

### Identification of DMS associated with nutritional history

Previously, an experiment was reported in which pregnant mice were subjected to undernutrition (U) during the last third of their pregnancy and this feeding regimen was continued during lactation generating the UU pups, alternative some mothers were changed to a control diet (C) during lactation and generated the UC pups. CC and CU protocols were also implemented to generate the respective pups. All pups were euthanized and their livers analyzed [23]. We used 16 liver samples derived from the same experiment: CC (n = 4), CU (n = 5), UC (n = 2) and UU (n = 5) to profile changes in methylation using the approach described in previous section.

### CpG distribution related to UCSC known genes

CpGs were classified according to their genomic location: 1) TSS region (−3 Kb to +2 Kb of the TSS); 2) gene body region; 3) 3′ end region encompassing 3 Kb of DNA sequences downstream of each transcriptional end; and 4) intergenic region. Coordinates for the beginning and end of these regions were taken from the mm9 building downloaded from the UCSC Genome Bioinformatics browser (http://hgdownload.cse.ucsc.edu/goldenPath/mm9/database/knownGene.txt.gz). The equally unmethylated CpGs (497,547) were defined as the CpGs with the average normalized *df* above the cutoff value of unmethylation in all four tissues and excluding any overlapping with DMSs.

### CpG distribution related to CGIs

CpGs were mapped to three regions relative to CGIs and their 2-kb flanking regions (shores), including inside CGI, in CGI shores, and outside of CGI and its 2 Kb shores. Five CGI definitions were used for this analysis [26]–[30].

### Measures of the association between TS-DMS and tissue-specific expressed genes

We used the Gene Expression Barcode 2.0 browser to obtain a list of genes expressed in brain, kidney, liver, and testis (http://rafalab.jhsph.edu/barcode/index.php?page=tissuegene). The original gene expression information in this database was extracted from publicly available gene expression results of 9,652 samples using the Mouse Genome 430 2.0 microarray [31]. In this set, a gene is considered expressed in a specific tissue if it is expressed in more than 95% of the samples of this tissue. We further extracted tissue-specific expressed genes by collecting, those that are expressed in only one of the four tissues. We considered a gene to be tissue differentially methylated when it has at least one TS-DMS within the region encompassing ±3 Kb from its edges. To standardize the different lists, all of them were converted to DAVID identifiers using the Gene ID conversion tool in NIAID's DAVID Bioinformatics Resources 6.7 [26], [27] website (http://david.abcc.ncifcrf.gov/). Only genes with DAVID identifiers were considered in this study. The odds ratio was calculated as the ratio of the odds of tissue-specific un-methylation occurring in tissue-specific expressed genes versus the odds of it occurring in non-tissue-specific expressed genes. Four binary sets of data were created to calculate the odds ratio (OR) in each tissue according to the formula: OR = (a/c)/(b/d). Where “a” is the number of genes tissue differentially methylated that were uniquely expressed in the same tissue; “b” are genes not associated with TS-DMS but uniquely expressed in the analyzed tissue; “c” is the number of genes tissue-differentially-methylated but expressed in more than one tissue; “d” is the number of genes not associated to TS-DMS and expressed in more than one tissue. Both ORs and p-values were calculated by using the logistic regression function in R package with one degree of freedom.

### Overlap between TS-DMS and published tissue-specific cis-regulatory sequences

We downloaded a list of 32,266 mouse tissue-specific enhancers in cortex, liver, kidney, and testis identified by H3K4me1 ChIP-Seq study (http://chromosome.sdsc.edu/mouse/download.html) [32]. We aligned our identified TS-DMSs and a total of 7 million surveyed CpGs in the genome to the enhancers based on their genomic locations. Enhancer was defined as being covered by our eMSCC method if it has at least one TS-DMS. To quantify the fold enrichment of TS-DMS in enhancers with respect to the genome, we calculated the ratio between [number of TS-DMS within enhancers/total number of TS-DMS] and [total number of surveyed CpGs within enhancers/total number of surveyed CpGs in the genome]. We estimated the significance of the enrichments by simulation, which allowed us to assign p values based on the distribution of the fold enrichments generated from simulation of 100,000 iterations. For each iteration we randomly generated a set of genomic regions with the same number of enhancers and the same size (bp) of each enhancer. In addition, we examined the overlap of TS-DMS with 15,435 active enhancers, which were defined by both H3K4me1 and H3K27ac marks.

### Functional annotation of CpGs using NIAID's DAVID Bioinformatics Resources

We used the tool provided in NIAID's DAVID Bioinformatics Resources 6.7 [26], [27] to analyze enrichments in five main categories: biological process, cellular component, molecular function, KEGG pathway, and tissue expression. We used the Bonferroni-corrected *P* value of 0.05 as the threshold to identify significantly enriched categories, which were sorted by -fold enrichment over the background frequency in the human genome. We focused on genes associated with CpGs uniquely unmethylated in one of the four tissues, using gene names derived from the Gene ID conversion tool within DAVID. A gene was defined as being associated with a CpG if the CpG was within 3 Kb upstream to 3 Kb downstream of the gene.

### Functional enrichment analysis

The functional annotation tables usually display a rank-order with broader general term at the top, and most specific terms toward the bottom. Although the upper terms have greater statistical support (largely enriched, o lower p-values), they are usually less to almost no informative, but generally are the reported terms when the main output of the enrichment tool is based on functional enrichments tables. DAG graph facilitates the systematic set up of thresholds to select relevant terms based on the structure of the results. We used Gene Ontology enRIchment anaLysis and visuaLizAtion tool (GORILLA) [33] which allows to identify the most informative terms that are significant enriched.

### Unsupervised hierarchical clustering analysis

Unsupervised hierarchical cluster analysis was performed with Cluster 3.0 and displayed using TreeView [34], [35].

## Results

### Identification of Tissue-Specific Differentially Methylated Sites (TS-DMS)

**Figure 1:**
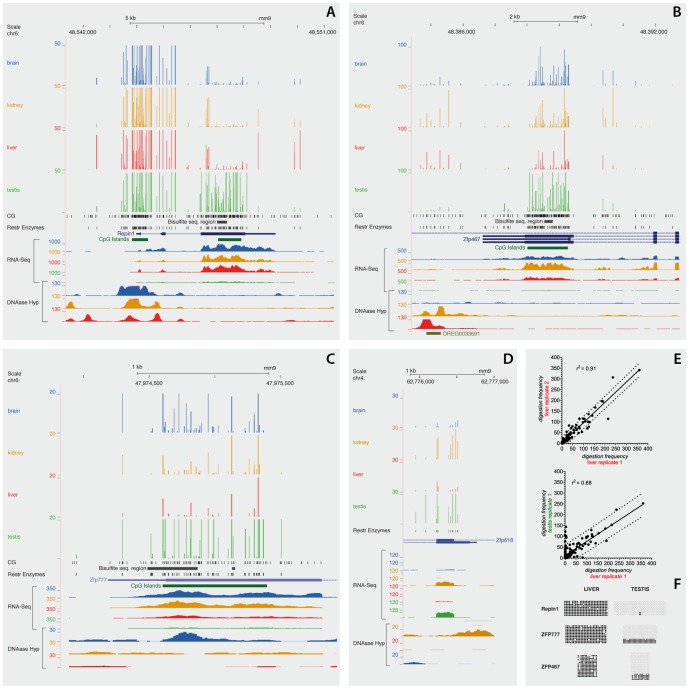
Identification of tissue differential methylated sites (T-DMS) led to the identification of tissue differentially methylated regions (T-DMR). Averaged digestion frequencies were profiled for each tissue and depicted with the UCSC genome browser. The long RNA-seq track generated by the transcriptome group at Cold Spring Harbor Laboratories and the Center for Genomic Regulation in Barcelona display the density of mapped reads. Individual tissues were harvested from 8-month C57BL/6J mice. The UW ENCODE group generated the DNaseI hypersensitivity track, the signal represent the density of reads mapped within a 150 bp sliding window. Tissues were harvested from mice of the same strain and age as described above. A) region containing the entire Repin1 gene. B), C) and D) regions spanning the last exon of the Zfp467, Zfp777 and Zfp618 genes respectively. E) scatter plot comparing digestion frequencies of liver replicates (upper panel) or different tissues (lower panel). Data was collected for the “chr6: 48,537,207–48,558,557” interval. The solid lines represent the result of a linear regression; the dashed lines defined the 95% interval of prediction. Data outside this interval could represent tissue differentially methylated CpGs. F) bisulfite sequencing analysis confirmed the discovered tissue-specific differentially methylated region. Regions selected for this analysis are shown as black bars in the panels A, B and C.

**Figure 2:**
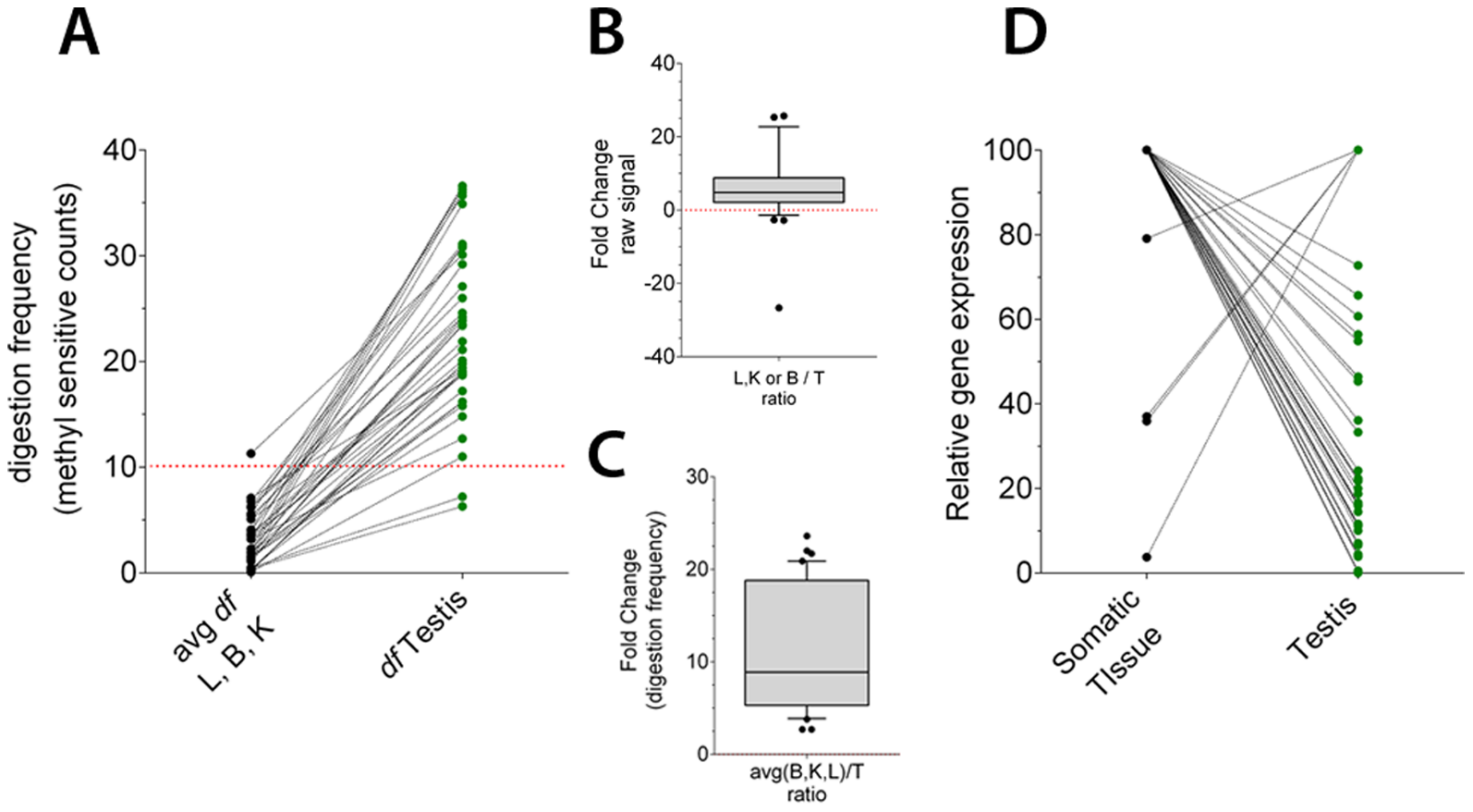
Co-occurrence of differential tissue gene expression and differential tissue methylation at 3′ terminal exons of a set of Zfp genes. A, paired comparisons of the digestion frequencies (somatic tissues vs. testis) tabulated in Table 1 under the column header: “methylation in 3′ exon”. B and C, distribution of linear fold change (somatic tissues vs. testis) values for gene expression and methylation respectively. Box plots represent the data tabulated under FCh columns in Table 1. D, paired comparison of gene expression (somatic tissues vs. testis), the somatic tissue with the highest raw signal was chosen for comparison in each pair.

Brain, kidney, liver and testis were dissected from three C57BL/6J mice and genomic DNA was isolated from every specimen. Each sample was treated with four methylation-sensitive restriction enzymes to compare the extent of digestion at approximately 7 million CpGs. Briefly, twelve CpG-tag libraries were prepared according to our previously published method [15], and deep sequenced to generate 37.5 to 51.4 million aligned reads per sample, Table S1 in Supporting Information S1, Data Set S1. These reads contain ∼27-bp sequences located adjacent to CCGG, ACGT, GCGC, CCGC and GGCG digested sites. Digestion frequencies (*df*) were calculated by counting the number of reads aligned to each site in the genome. A site with low *df* may indicate either a need for deeper sequencing or that the site is highly methylated. Three observations support the idea that most poorly digested sites were associated with high levels of methylation, rather than with poor coverage or random sampling of CpG-tags during sequencing: 1) Of the approximately 7 million CpGs tested across this study, 5,283,360 were represented in the libraries suggesting an effective coverage; 2) To a large extent, *sites* that were resistant to the enzymatic digestion, were arranged in tandem within the body of genes, repetitive sequences and intergenic regions. All these places are known to harbor most of the genomic methylation marks; 3) Digestion frequencies for poorly identified sites were consistently low in all the tissues and in all the replicates, which is not expected as a result of random sampling. Figure 1 illustrates these observations. Each digestion profile represents the averaged frequencies of three replicates. Highly digested *sites* segregate from those resistant to the enzymes. The majority of *sites* located in the 5′ regions of genes and in CGIs scored high *digestion frequencies*, whereas sites in intergenic regions, repetitive DNA or gene bodies, scored low. Figure 1 shows tissue specific differentially methylated regions (TS-DMR) spanning the entire intragenic CGIs in the last exon of several Zinc finger proteins, Table 1. All these differentially methylated CGIs distinguish somatic tissues from testis. Indeed, this epigenetic dichotomy seems to reflect a transcriptional dichotomy, Figure 2. For instance, the CGI located in the 5′ region of the Repin 1 gene is un-methylated and coincides with DNAse hypersensitivity in all the somatic tissues, whereas, the intragenic CGI located in the 3′ region of the gene is hyper-methylated, and insensitive to DNase I.

**Table 1:** A set of tissue differentially expressed Zfp genes with TS-DMS in their 3′ terminal exon.

				Methylation in 3′ exon^1^					Gene expression: RNA-seq^2^				
Gene List	5′ CGI?^3^	3′ exon CGI?	DMR in 3′ exon	B	K	L	T	FCh^4^	B	K	L	T	FCh^5^
Zfp92	no	yes	chrX:70,667,009–70,668,669	1.8	1.8	2.4	15.8	7.8	25	1	1	5	5
Zfp787	yes	yes	chr7:6,083,186–6,085,905	6.7	8.3	4.0	30.8	4.9	343	814	550	181	4.5
Zfp775	no	yes	chr6:48,569,295–48,571,527	3.1	2.0	1.7	36.6	16.2	140	168	62	56	3
Zfp768	yes	yes	chr7:134,486,685–134,488,364	2.6	7.4	5.3	27.1	5.3	313	1425	462	782	1.8
Zfp689	yes	yes	chr7:134,587,450–134,589,790	6.3	9.6	4.4	31.1	4.6	148	138	43	412	−2.8
Zfp647	yes	yes	chr15:76,741,187–76,743,420	1.0	0.8	1.5	23.9	21.7	80	34	15	9	8.9
Zfp64	yes	yes	chr2:168,751,178–168,753,416	7.5	2.3	1.3	21.9	5.9	289	249	159	46	6.3
Zfp629	no	yes	chr7:134,754,156–134,757,445	2.1	2.9	0.6	36.0	19.1	235	275	131	200	1.4
Zfp536	yes	yes	chr7:38,264,240–38,266,631	4.1	4.0	2.7	21.1	5.9	397	3	2	28	14.2
Zfp467	yes	yes	chr6:48,387,913–48,389,959	14.8	12.0	7.0	30.1	2.7	251	605	235	27	22.4
Zfp398	yes	yes	chr6:47,816,064–47,817,270	0.6	0.4	0.2	6.3	6.3	300	315	87	146	2.2
Zfp358	yes	yes	chr8:3,494,937–3,497,650	1.5	1.3	1.4	29.2	20.9	901	1187	329	47	25.3
Zfp324	yes	yes	chr7:13,555,845–13,557,613	3.0	4.1	2.6	36.6	11.4	97	212	108	14	15.1
Zfp282	yes	yes	chr6:47,853,767–47,857,203	8.9	8.0	4.3	19.1	2.7	279	159	49	28	10.0
Zfp275	yes	yes	chrX:70,599,182–70,599,994	1.0	0.3	0.3	7.2	7.2	125	105	52	58	2.2
Zfp213	yes	yes	chr17:23,694,614–23,695,442	3.1	4.3	2.3	12.7	3.9	123	256	124	62	4.1
Zfpm2	yes	no	chr15:40,933,302–40,935,151	1.1	1.0	1.5	23.5	19.6	108	12	4	39	2.8
Zfp94	yes	no	chr7:25,087,544–25,089,206	1.4	0.9	1.0	24.2	22.0	97	31	15	44	2.2
Zfp809	yes	no	chr9:22,043,700–22,045,660	0.2	0.3	0.2	19.8	19.8	239	180	158	52	4.6
Zfp804a	yes	no	chr2:82,099,024–82,099,872	2.9	12.4	3.2	24.2	3.9	181	1	1	1	181
Zfp791	no	no	chr8:87,633,541–87,638,183	1.6	1.6	1.3	14.8	10.1	470	526	544	307	1.8
Zfp780b	no	no	chr7:28,746,705–28,746,943	6.3	7.1	5.2	26.0	4.2	52	15	16	6	8.7
Zfp764	yes	no	chr7:134,548,323–134,549,900	2.7	2.8	1.4	23.4	10.2	104	154	46	6	25.7
Zfp691	no	no	chr4:118,842,688–118,844,133	2.9	4.3	2.3	20.1	6.4	120	379	230	479	−1.3
Zfp69	yes	no	chr4:120,602,876–120,604,472	1.5	2.1	0.8	34.9	23.6	28	14	3	17	1.6
Zfp672	yes	no	chr11:58,129,068–58,131,818	7.9	6.1	2.9	35.7	6.3	545	1083	424	711	1.5
Zfp668	yes	no	chr7:135,009,556–135,012,952	1.9	1.8	0.9	16.2	10.6	188	74	55	42	4.5
Zfp619	yes	no	chr7:46,790,771–46,795,803	0.3	0.4	0.4	18.8	18.8	43	21	9	5	8.6
Zfp612	yes	no	chr8:112,612,925–112,614,371	0.3	0.3	0.3	19.0	19.0	350	31	19	56	6.3
Zfp608	yes	no	chr18:55,057,322–55,060,683	4.2	7.2	3.8	19.4	3.8	101	86	19	273	−2.7
Zfp42	no	no	chr8:44,380,959–44,381,843	0.8	1.6	2.4	17.2	10.9	3	1	2	80	−26.7
Zfp341	yes	no	chr2:154,471,197–154,472,435	4.8	5.1	2.0	30.9	7.8	131	53	41	19	6.9
Zfp27	no	no	chr7:30,679,004–30,681,841	0.1	0.1	0.0	11.0	11.0	198	46	36	32	6.2
Zfp248	yes	no	chr6:118,388,618–118,389,661	0.4	0.3	0.4	16.2	16.2	12	2	1	2	6
Zfp239	no	no	chr6:117,820,865–117,823,017	3.3	4.7	8.2	24.6	4.6	675	76	2	109	6.2
Zfp180	yes	no	chr7:24,889,403–24,892,210	1.8	2.0	2.1	19.0	9.7	448	203	88	84	5.3
Zfp112	no	no	chr7:24,902,263–24,904,868	4.6	3.2	2.7	18.7	5.3	36	6	2	4	9
Zfp365	yes	no	chr10:67,350,323–67,351,704	4.8	3.9	3.5	36.3	8.9	3983	8	1	3	1327.7
Zfp809	yes	no	chr9:22,043,950–22,044,900	0.2	0.3	0.2	19.8	19.8	239	180	158	52	4.6

1 ) The level of methylation in the 3′exon of each Zfp gene was calculated for each tissue by adding the averaged digestion frequencies of triplicates at each CpG identified in the exon, and dividing by the number of identified CpGs in the exon. A digestion frequency around 26 is typically found in un-methylated CGI (Figure S2E in Supporting Information S1), where *df*<10 are associated to methylated CGI.

2 ) The numbers represent the raw signal for the density of mapped reads (wiggle format) in the CSHL Long RNA-seq track in the UCSC genome browser (NCBI37/mm9 assembly).

3 ) All 5′ CGI were found un-methylated in all the tissues for each Zfp gene listed in this table.

4 ) Fold changes calculated as the ratio between *df* at testes and the *avg df* of the three somatic tissues.

5 ) Fold change is calculated as the ratio of somatic tissue vs testes. The somatic tissue with the highest RNA-seq signal or “*df*” was chose in each case. Abbreviation: CGI for CpG Island, *df* for digestion frequency, B for brain, K for kidney, L for liver and T for testes.

Whilst these results uphold the effectiveness of **M**ethyl-**S**ensitive **C**ut **C**ounting (MSCC) to profile methylation [15], [36], [37], we harnessed the power of this method to perform a multiple testing procedure to detect differentially methylated sites (DMS), [38]. By implementing MSCC in a multiple pairwise comparison design, we controlled site-specific biases, Figure S1 in Supporting Information S1. In addition, the variability introduced by random errors is weighed in the denominator of the equation used to calculate the t-statistic; this allows us to ascribe the differences in the digestion frequencies to differences in methylation levels.

Although our experimental design allows for comparisons across the dynamic range, we contrasted only those sites that are highly supported by sequencing data. To conduct this selection we first normalized the libraries to reach similar sequencing depth Figure S2E in Supporting Information S1. The average *df* was computed for every CGI defined by the Takai and Jones algorithm [26]. For each library, the 21,246 computed scores were organized in a frequency histogram, Figure S2E in Supporting Information S1. The figure shows a bimodal distribution that is consistent with the bimodal pattern of methylation found in CpG islands [39]. As such the frequency histogram has been used to directly determine the methylation status of individual CGIs [15], but here we used them to derive an unrefined estimate of the relationship between *digestion frequency* and rate of methylation at each surveyed CpG. The *sites* with a *df* of 10 or less were considered heavily methylated and *sites* not reaching the cut-off in any of the libraries were excluded. For the identification of DMS multiple pairwise Welch t tests were performed. The null hypothesis was stated as no difference between the averages of the triplicates. Based on the differences detected by the Welch t-test further directional decisions were made to sort the means according to their values. This allowed the identification of *sites* whose rate of digestion was unusually high or low in only one of the tissues. These *sites* were called TS-DMS. The test was designed to control for errors associated with wrong rejections of the null hypothesis and also for assigning wrong inequality patterns. A mixed directional false discovery rate (mdFDR) of 10% was chosen in this analysis [25]. Mouse gDNA samples were spiked with lambda gDNA (λDNA) to achieve an equimolar ratio between both genomes. In this way, for each one of the 1,202 completely *un-methylated sites* present in the lambda genome, 12 replicates were obtained, Data Set S2. Figure S2 A and C in Supporting Information S1 suggest that *df* computed for these replicates are approximately normally distributed with very slight departures in the tails of the distribution. Comparison of *digestion frequencies* among λ-gDNA replicates reveals that 10% of the 1,202 λ-sites were found to have significant differences in their *digestion frequencies*. Although no differences were expected between the 12 un-methylated λDNA replicates, our results show that these empirical values for false discovery rate were very close to the theoretical value of 10% selected for this study.

**Figure 3:**
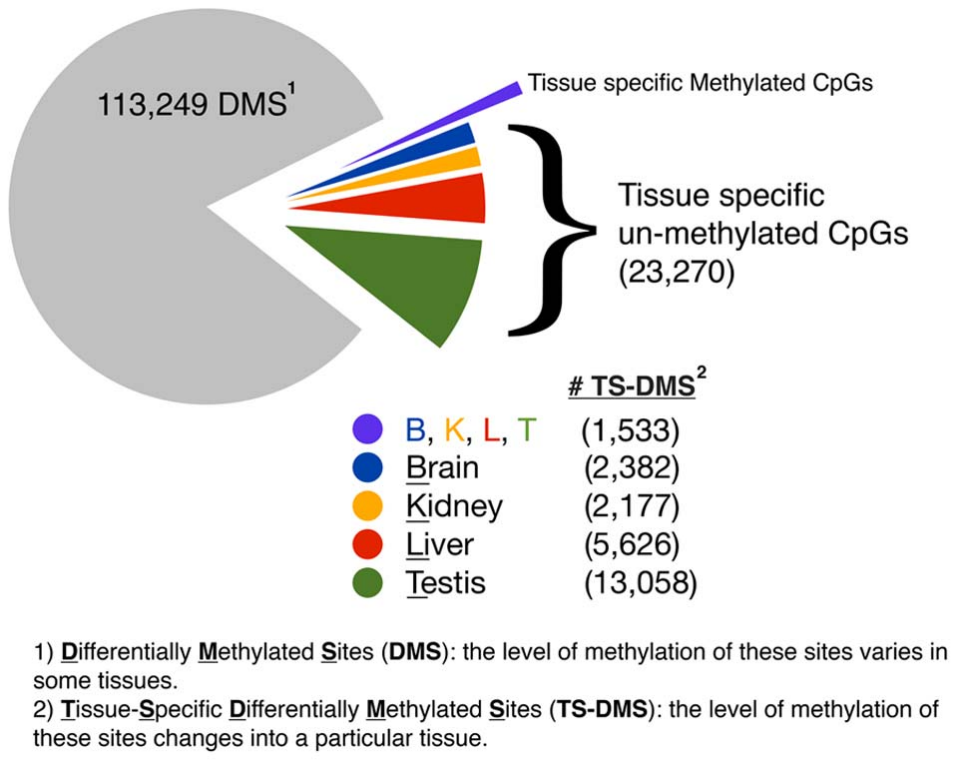
Summary of the findings of differential methylation observed for the comparison of tissue. Of the 138,052 sites with differentially methylated marks, called DMS, only 24,803 were also TS-DMS, defined here as CpGs uniquely methylated or un-methylated in one but not in the other tissues. The remaining 113,249 sites are just DMS, meaning that methylation at these CpGs varies at a detectable level among compared tissues.

Applied to CpGs from the mouse genome the comparative differences reveals 150,428 sites where the methylation varies to the same extent among the analyzed four tissues. Furthermore, retaining only the sites whose *digestion frequencies* differed by a minimum of 10 resulted in a final count of 138,052 DMS (Data Set S3). Only 24,803 of these DMS were TS-DMS, i.e. sites that were hypo or hyper-methylated in only one tissue, Figure 3. Notably, 23,270 (94%) of these TS-DMS are un-methylated CpGs in one of the four tissues, of which 57% were in the testis.

### Distribution of TS-DMS in relation to Genes and CpG Islands

**Figure 4:**
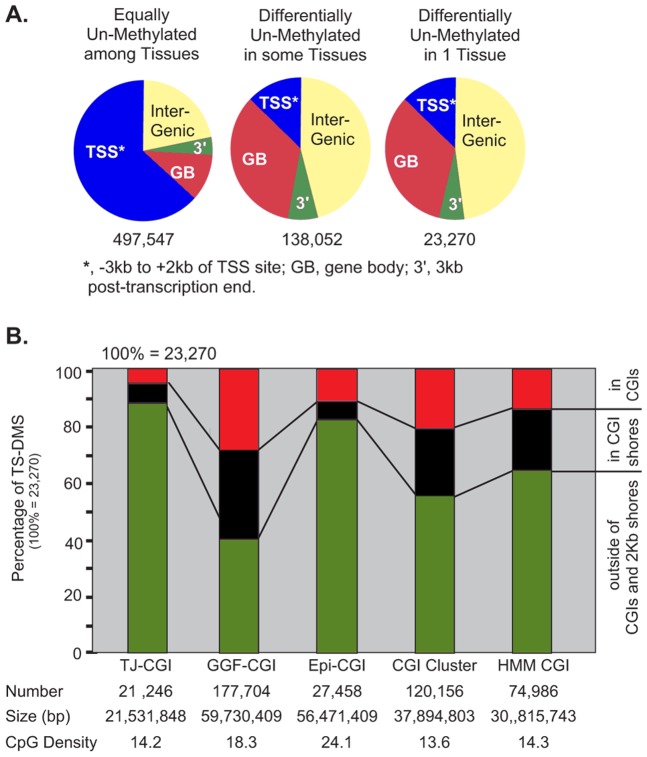
Distribution of TS-DMS relative to promoters, CGI, CGI shores, experimentally determined un-methylated regions (UMR) or shores of UMRs. A) Distribution of equally and tissue differentially un-methylated CpGs relative to UCSC genes. B) Distribution of differentially un-methylated CpGs relative to CGIs and their 2-kb shores. TJ-CGIs (Takai and Jones CpG islands), GGF-CGIs (Gardiner- Garden and Frommer's CpG islands), Epi-CGIs (epigenetically predicted CpG islands), CGI clusters (CpG clusters), HMM CGI (Hidden Markov Model predicted CpG islands).

An overwhelming number of genome-wide methylation studies focused on CGIs and promoter regions have proposed them as hot spots for epigenetic control of gene expression through CpG methylation [40]. To investigate if our results are consistent with this concept, we compared the distribution of *sites* having a comparable methylation level among the four tissues with the distribution of TS-DMS. Almost all the *sites* identified as DMS or TS-DMS were found in introns or intergenic regions which are remote from promoters, Figure 4A. Recently, it has been proposed that intergenic and intragenic CGIs function as alternative promoters which can be repressed in a tissue specific manner through methylation of their CpGs [11], [12], [14]. However we found that only 5 to 25% of TS-DMS were parts of CpG islands. The fraction of TS-DMS in CGIs increased proportionally to the number of island in each set, Figure 4-B.

Most recently, CGI shores, not CGI, have been proposed to be the targets of tissue-specific epigenetic regulation [41], [42]. We found that the fraction of TS-DMS mapping to CGI shores (2000 base pairs from the edge of a CGI) follows the same trend and proportions than those mapping to CGIs, Figure 4B. The different CGI sets used in this study represent efforts to improve predictions of functionality on GC-rich genomic sequences, mainly promoter activity and un-methylated status. Notably the Gardiner- Garden and Frommer's set (GGF-CGI), which is the oldest, biggest and considered the less rigorous in term of functional prediction, is the one that collects the biggest number of TS-DMS. Approximately 60% of the tissue specific un-methylated CpGs are equally distributed between GGF-CGI and their shores. Conversely, the GGF-CGI-derived more stringent sets, such as those produced applying the Takai and Jones criteria [26] or with an epigenome prediction pipeline [28], are the ones containing the smallest fractions of TS-DMS in their CGIs or shores, Figure 4b. Thus the main characteristic in a set correlating with the proportion of TS-DMS is their number of predicted CpG rich loci, suggesting that the effort done to improve the prediction of epigenetically regulatory sequences in a genome failed to capture the DNA features that guide tissue-specific epigenetic mechanisms.

### Association between TS-DMS and tissue-specific expressed genes

The Gene Expression Barcode browser contains a list of genes whose levels of expression, in 89 murine tissues, were represented as binary calls (expressed or not-expressed) [31]. These lists were made from 9,652 publicly available gene expression results obtained with the Mouse Genome 430 Array 2.0. Genes detected above threshold in more than 95% of samples of a specific tissue are considered expressed in that tissue. We considered a gene to be expressed in a tissue-specific manner when it is expressed in only one of the four organs included in our study. In addition we created a list including all the genes with at least one TS-DMS in the region extending from -3 Kb to +3 Kb of the edges of the gene. We found that genes associated with TS-DMS are more likely to be expressed in a tissue specific manner. The strength of this association varies across the different tissues, Table 2.

**Table 2:** Association of genes with TS-DMS and tissue specific gene expression in each tissue.

Tissue	TS-DMS associated genes	TS-expressed genes^1^	Overlaps	Odds Ratio^2^	p-value^3^
Brain	1,035	1,080	160	3.18	7.42E-36
Kidney	940	434	52	2.55	7.41E-10
Liver	2,090	878	339	5.5	4.64E-118
Testis	2,700	2,393	438	1.31	1.97E-06

1 ) Taken from the Gene Expression Barcode 2.0 database (http//:rafalab.jhsph.edu/barcode/).

2 ) The ratio of the odds of tissue-specific un-methylation occurring in tissue-specific expressed genes.

3 ) p-values calculated using the logistic regression function in R package with one degree of freedom.

### TS-DMS are more frequently found at CpGs inside tissue specific cis-regulatory sequences

A map of cis-regulatory elements for the mouse genome has recently been published [32]. The map includes 53,834 putative promoters and 234,764 potential enhancers, many of which are tissue specific. These enhancers were defined according to the presence of ChIP-Seq peaks for histone H3 lysine 4 mono-methylation marks (H3K4me1) outside promoters. We obtained the coordinates for 24,128 tissue-specific enhancers that are divided between liver, kidney and testis (http://chromosome.sdsc.edu/mouse/download.html). We compared the relative number of TS-DMS and surveyed CpG sites inside and outside of these enhancer-DNA sequences. We found that the percentage of TS-DMS within enhancers is up to 30 times higher than outside enhancers Table 3 (p-value<10^−5^, simulation of 100,000 iterations), with enrichment varying across tissues. Next we examined the overlap of TS-DMS with active enhancers, as defined by acetylation marks (H3K27ac), [32] (Table 4). A comparison of the results (Table 3 with Table 4) shows that most of the TS-DMS derived from somatic tissues are localized in active enhancers, while the opposite is true for the case of the TS-DMS derived from the testis. For example, of the 792 TS-DMS marking enhancers in liver, 561 (70%) are in active enhancers.

**Table 3:** Overlap between TS-DMS and tissue-specific enhancers derived from ChIP-Seq H3K4me1 peaks (poised and active enhancers).

Tissue	TS-enhancers	TS-DMSs	Overlaps^1^	# CpG^2^	Fold Enrichmen^3^	P-value^4^
**Cortex-Brain** 5	8,138	1,149	60; (5.2%)	64,512	5.63	<1.0 E-5
**kidney**	5,976	1,115	147 (13.2%)	44,549	20.58	<1.0 E-5
**liver**	8,701	2,933	792 (27.0%)	63,053	29.79	<1.0 E-5
**testis**	9,451	8,865	3,314 (37.4 3,314 (37.4%)	164,862	15.77	<1.0 E-5

1 ) Number of TS-DMS within TS-enhancer regions.

2 ) Number of surveyed CpGs within TS-enhancer regions.

3 ) The quotient of two ratios (TS-DMS within TS-enhancer regions/total number of TS-DMSs) and (total number of surveyed CpGs within TS-enhancer regions/total number of surveyed CpGs in the genome).

4 ) p-value calculated according to the distribution of the fold enrichments generated from simulation of 100,000 iterations.

5 ) While these enhancers are derived from cortex the TS-DMS are derived from whole brain.

**Table 4:** Overlap between TS-DMSs and active enhancers (H3K27ac and H3K4me1 marks).

Tissue	TS-enhancers	TS-DMSs	Overlaps^1^	# CpG^2^	Fold Enrichment^3^	p-value^4^
**Cortex-Brain** 5	4,128	1,149	43; (3.7%)	33,010	7.89	<1 E-5
**kidney**	3,721	1,115	115 (10.3%)	29,187	24.58	<1 E-5
**liver**	4,941	2,933	561 (19.1%)	38,697	34.38	<1 E-5
**testis**	2,645	8,865	758 (8.6%)	50,783	11.71	<1.E-5

1 ) Number of TS-DMSs within enhancers with H3K27ac marks.

2 ) Number of surveyed CpGs within enhancers with H3K27ac marks.

3 ) The quotient of two ratios, (number of TS-DMS within H3K27ac enhancers/total number of TS-DMS) and (total number of surveyed CpGs within H3K27enhancers/total number of surveyed CpGs in the genome).

4 ) p-value calculated according to the distribution of the fold enrichments generated from simulation of 100,000 iterations.

5 ) While these enhancers are derived from cortex the TS-DMS are derived from whole brain.

### Genes associated with TS-DMS are enriched in functional annotations specific to the corresponding tissue

**Figure 5:**
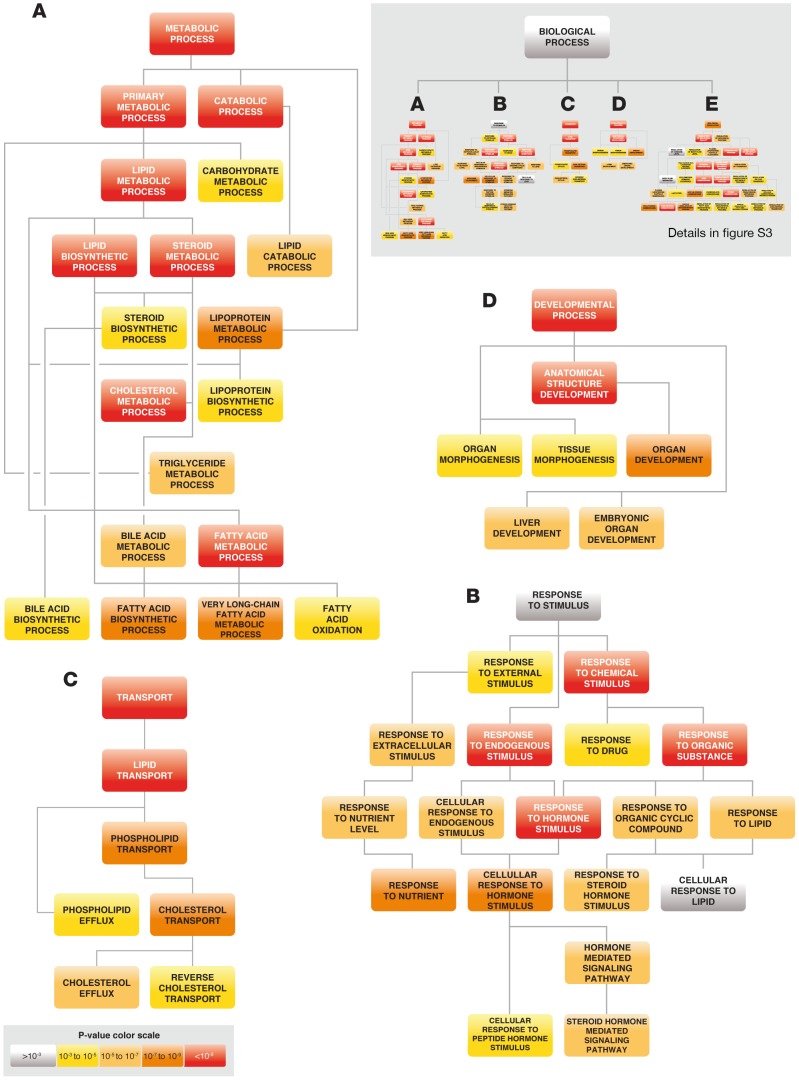
DAG graphical representation of the functional profiles extracted from genes associated to liver specific un-methylated sites. 2500 genes directly linked to liver specific TS-DMS were input in the Gorilla tool. Enriched GO terms are depicted using a direct acyclic graph with color code reflecting the statistical support of their enrichment. Most significant branches whit not redundant nodes are shown separately for enhanced detail.

We created two lists representing different levels of association between the TS-DMS and their corresponding genes, Data Set S4. If a TS-DMS is outside the region occupied by the gene ±3,000 bp it was assigned to the nearest TSS (weak association). If a TS-DMS is inside the region occupied by the gene ±3,000 bp it was directly assigned to that gene (strong association). Gene annotation enrichment analyses using these gene lists were mainly based on the Gene Ontology data base (GO) [43]. To obtain the functional profiles we used DAVID Bioinformatics Resources [44], [45] and Gene Ontology enRIchment anaLysis and visuaLizAtion tool (GORILLA) [33]. Genes associated with TS-DMS were significantly enriched in tissue-specific expression annotation in each of the four tissues, (Table 5). Table 6 tabulates the functional enrichment results for biological processes in the context of a directed acyclic graph (DAG) structure created using the *GOrilla* tool, e.g. Figure 5. The table includes all the terms at terminal nodes of the DAG describing the most specific and informative biological processes that showed significant enrichment. Terms described in these sets matched distinct attributes of the physiology and biochemistry of each particular tissue, Figures 5 and Figures S3and S4 in Supporting Information S1, also Table 6. Interestingly, genes physically linked to TS-DMS are mostly associated with typical activities of the respective adult organ, whilst many genes with TS-DMS distal to their promoters are associated with embryonic development, Table 6 and Figure S4 in Supporting Information S1. Finally, we scored and ranked the genes according to the number of TS-DMS associated with them, and used the *GOrilla* tool to discover GO terms that are significantly enriched at the *top* of a ranked gene list. In the case of liver, we found very high enrichments for genes involved in lipid metabolism and homeostasis, particularly for processes including synthesis, transport and cellular response to cholesterol, Table 7.

**Table 5:** Enrichment of tissue-specific gene expression annotations performed with the DAVID Bioinformatics Resources.

	TS-DMS^1^	TS-DMS in genes^2^	Genes with TS-DMS	TS-genes^3^	%	p-value^4^
Brain	2382	1402	1098	575	52	5.6 E-36
Kidney	2177	1250	999	170	17	7.6 E-8
Liver	5626	3326	2218	702	32	1.1 E-79
Testes	13058	5848	3042	598	20	3.8 E-5

1 ) Number of CpGs that were identified as uniquely un-methylated.

2 ) Number of TS-DMS located in the region spanned from ±3 kb of the gene edges.

3 ) Genes annotated as tissue-specific expressed.

4 ) Adjusted p-values (Bonferroni correction for family-wise error rate).

**Table 6:** Gene Ontology enrichment results on the list of genes associated to liver specifically un-methylated CpG.

GO Term^1^	Description	P-value	FDR q-value	Enrichment	B^2^	b^3^
**Liver**						
GO:0042632	cholesterol homeostasis	1.79E-13	4.42E-11	5.31	48	25
GO:0008203	cholesterol metabolic process	5.52E-13	1.14E-10	3.94	88	34
GO:1901606	alpha-amino acid catabolic process	2.17E-10	3.05E-08	4.11	62	25
GO:0007169	transmembrane receptor protein tyrosine kinase signaling pathway	3.30E-10	4.56E-08	2.39	243	57
GO:0007584	response to nutrient	3.64E-08	3.56E-06	4.27	43	18
GO:0070328	triglyceride homeostasis	4.60E-08	4.35E-06	6.11	20	12
GO:0006633	fatty acid biosynthetic process	4.94E-08	4.60E-06	2.91	105	30
GO:0046889	positive regulation of lipid biosynthetic process	1.08E-07	9.55E-06	3.71	55	20
GO:0090207	regulation of triglyceride metabolic process	1.26E-07	1.10E-05	5.3	25	13
GO:0033344	cholesterol efflux	1.95E-07	1.61E-05	5.56	22	12
Brain						
GO:0007399	nervous system development	1.30E-08	2.26E-06	2.69	307	40
GO:0007411	axon guidance	1.71E-08	2.76E-06	3.66	141	25
GO:0007409	axonogenesis	1.98E-08	3.11E-06	3.75	132	24
GO:0007169	transmembrane receptor protein tyrosine kinase signaling pathway	8.11E-08	1.07E-05	2.81	242	33
GO:0070588	calcium ion transmembrane transport	7.17E-07	7.43E-05	3.87	96	18
GO:0071902	positive regulation of protein serine/threonine kinase activity	1.20E-06	1.20E-04	2.81	198	27
GO:0007163	establishment or maintenance of cell polarity	2.00E-06	1.89E-04	3.98	83	16
GO:0006813	potassium ion transport	2.18E-06	2.04E-04	3.21	135	21
GO:0007626	locomotory behavior	2.94E-06	2.56E-04	2.97	160	23
GO:0050772	positive regulation of axonogenesis	3.47E-06	2.95E-04	4.95	50	12
Testes						
GO:0071805	potassium ion transmembrane transport	1.63E-08	5.94E-06	2.82	86	32
GO:0006816	calcium ion transport	3.98E-07	1.05E-04	2.08	175	48
GO:0007156	homophilic cell adhesion	5.06E-07	1.30E-04	2.78	71	26
GO:0007411	axon guidance	5.15E-07	1.30E-04	2.21	141	41
GO:0007126	meiosis	1.89E-06	3.95E-04	2.47	89	29
GO:0070192	chromosome organization involved in meiosis	2.27E-06	4.67E-04	3.67	31	15
GO:2000310	regulation of N-methyl-D-aspartate selective glutamate receptor activity	3.17E-06	6.09E-04	6.07	10	8
GO:0060079	regulation of excitatory postsynaptic membrane potential	4.53E-06	7.89E-04	3.22	40	17
GO:0032673	regulation of interleukin-4 production	8.87E-06	1.34E-03	3.96	23	12
GO:0007283	spermatogenesis	7.38E-05	7.66E-03	2.05	267	58
Kidney						
GO:0030029	actin filament-based process	9.49E-05	8.38E-03	2.35	232	24
GO:0006820	anion transport	6.78E-05	6.33E-03	2.16	316	30
GO:0022610	biological adhesion	3.49E-07	9.86E-05	2.03	639	57
GO:0007167	enzyme linked receptor protein signaling pathway	6.11E-05	5.81E-03	2.06	376	34
GO:0046847	filopodium assembly	4.26E-07	1.18E-04	8.53	24	9
GO:0016570	histone modification	5.33E-05	5.33E-03	2.39	238	25
GO:0042490	mechanoreceptor differentiation	2.55E-05	2.98E-03	6.27	29	8
GO:0030512	negative regulation of transforming growth factor beta receptor signaling pathway	1.05E-05	1.47E-03	5.41	42	10
GO:0009887	organ morphogenesis	5.03E-06	7.89E-04	2.2	393	38
GO:0035335	peptidyl-tyrosine dephosphorylation	5.82E-05	5.68E-03	4.99	41	9
GO:0043065	positive regulation of apoptotic process	3.30E-05	3.80E-03	2	443	39

1 ) GO terms at the terminal nodes of the DAG, p-values<0.0001 and enrichments bigger than 2 are displayed in the table.

2 ) “B” is the total number of genes in the background dataset annotated with GO term in row.

3 ) “b” is the number of genes in the ‘experimental set’ that are associated with the GO term in row.

**Table 7:** Significantly enriched GO terms at the top of a ranked gene list^1^.

GO Term	Description	P-value	FDR q-value	Enrichment	B^2^	b^3^
GO:0071396	cellular response to lipid	5.33E-07	3.36E-03	18.49	37	7
GO:0042592	homeostatic process	3.14E-06	9.88E-03	6.52	165	11
GO:0048878	chemical homeostasis	4.75E-06	9.97E-03	7.29	134	10
GO:0032844	regulation of homeostatic process	6.01E-06	9.46E-03	45.26	38	4
GO:0033993	response to lipid	6.62E-06	8.34E-03	10.29	76	8
GO:0034383	low-density lipoprotein particle clearance	8.32E-06	8.73E-03	27.74	5	4
GO:0055088	lipid homeostasis	1.40E-05	1.26E-02	15.85	37	6
GO:0009755	hormone-mediated signaling pathway	2.15E-05	1.70E-02	4.02	23	13
GO:0034381	plasma lipoprotein particle clearance	2.45E-05	1.72E-02	17.34	10	5
GO:0030301	cholesterol transport	2.55E-05	1.61E-02	13	16	6
GO:0015918	sterol transport	2.55E-05	1.46E-02	13	16	6
GO:0055092	sterol homeostasis	2.70E-05	1.42E-02	13.95	25	6
GO:0042632	cholesterol homeostasis	2.70E-05	1.31E-02	13.95	25	6
GO:2000021	regulation of ion homeostasis	4.76E-05	2.14E-02	71.67	18	3
GO:0043691	reverse cholesterol transport	4.97E-05	2.09E-02	61.43	7	3
GO:0065008	regulation of biological quality	7.28E-05	2.87E-02	4.04	290	12

1 ) 4,265 genes were ranked according to their contain in TS-DMS. The Gorilla web-based application was used to identify enriched GO terms at the top of the ranked list of genes.

2 ) “B” is the total number of genes in the background dataset annotated with GO term in row.

3 ) “b” is the number of genes in the ‘experimental set’ that are associated with the GO term in row.

### TS-DMS constitute a unique epigenetic signature of an adult mouse liver

**Figure 6:**
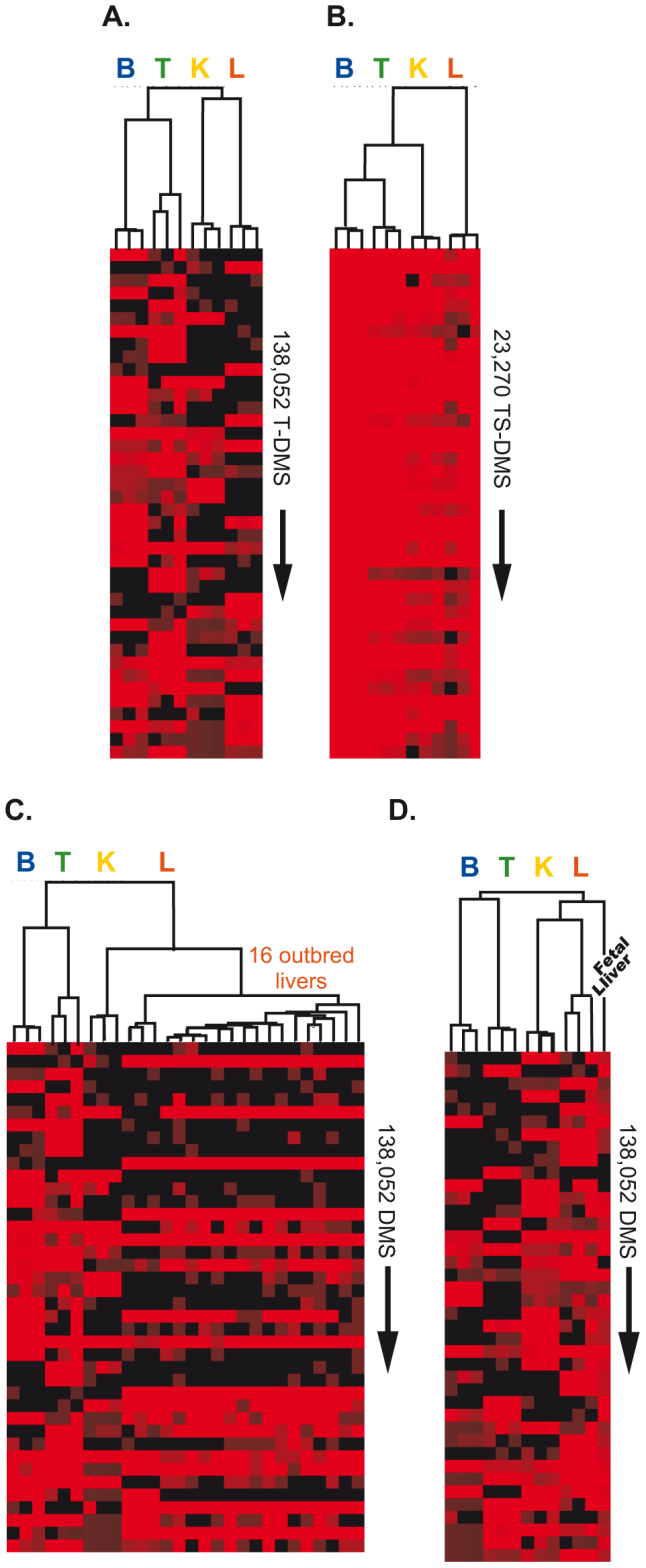
Epigenetic distances between tissues and biological replicates are assessed with unsupervised cluster analysis. The length of the branches in the hierarchical dendrogram measures the person correlation distances between digestion frequencies scored by CpGs identified as spot of tissue differential methylation. Shown are heat maps of the log2-transformed *digestion frequencies* (red: more than 50 reads per CpG, i.e., un-methylated; black: 0 reads per CpG, i.e., methylated). Rows represent the first 40 CpGs in the final cluster; each column represents one of the 12 tissue samples. A) 138,052 T-DMS. B) 23,270 TS-DMS (only the tissue specific un-methylated sites were clustered). C) MSCC data produced from livers of 16 outbred mice were combined with MSCC data of the 12 tissue samples. D) MSCC data produced from a liver of a C57BL/6J 12.5 d embryo was combined with MSCC data of the 12 tissue samples.

We performed an unsupervised hierarchical clustering analysis to visualize the TS-DMS structure between and within tissue samples as a hierarchical dendrogram where the branch lengths represent the degree of epigenetic similarity between samples. Samples from the same tissues were grouped together regardless of whether DMS or TS-DMS dataset was used (Figure 6 A and B). These results suggest that the DMS identified among 6,955,111-surveyed CpGs have the potential to function as tissue-specific methylation fingerprints.

We next tested if the liver specific DMS can be used as a unique epigenetic signature of the adult mouse liver. We combined the DMS found in the four-tissue dataset with similar data obtained from livers of 16 outbred mice and also the liver from a E11.5 C57BL/6J embryo. Despite the differences in the genetic background among these samples, the livers from all 19 mice co-clustered separately from brain, kidney, and testis, Figure 6C. A sample derived from embryonic liver (E11.5) was most similar to the adult liver samples than those from other organs, but still clustered separately from adult-stage liver, Figure 6D.

### A set of DMS derived from mice livers can trace nutritional history

Given the critical role of the liver in controlling whole-body metabolism, and the high frequency of TS-DMS in genes that control key aspects of the hepatic lipid homeostasis Figure S3 in Supporting Information S1, we analyzed the impact of prenatal and postnatal nutrient availability on liver methylation patterns. Differentially methylated sites (DMS) were isolated from mice whose mothers had been subjected to four different feeding protocols [23]: CC, prenatal and postnatal control diet; CU, prenatal control diet and postnatal food restriction; UU, prenatal and postnatal food restriction; UC, prenatal food restriction and postnatal control diet (for details see [23]). Comparison of CC, CU and UU liver samples led to the identification of 5,574 DMS (mdFDR 15%), Data Set S5. Practically all these DMS were hypo-methylated in mice that were exposed to a control diet during the whole experiment (CC) but hyper-methylated in those mice exposed to any of the diets including food restriction (UC, CU or UU). Out of these 5,574 DMS induced for food restriction, 533 were previously identified as differentially methylated sites in the comparisons among the four tissues (*vide supra*). Remarkably genes associated with these 533 DMS were highly enriched (between 4 to 15 times) in annotations related to SMAD protein signal transduction, steroid and fatty acid metabolic process.

**Figure 7:**
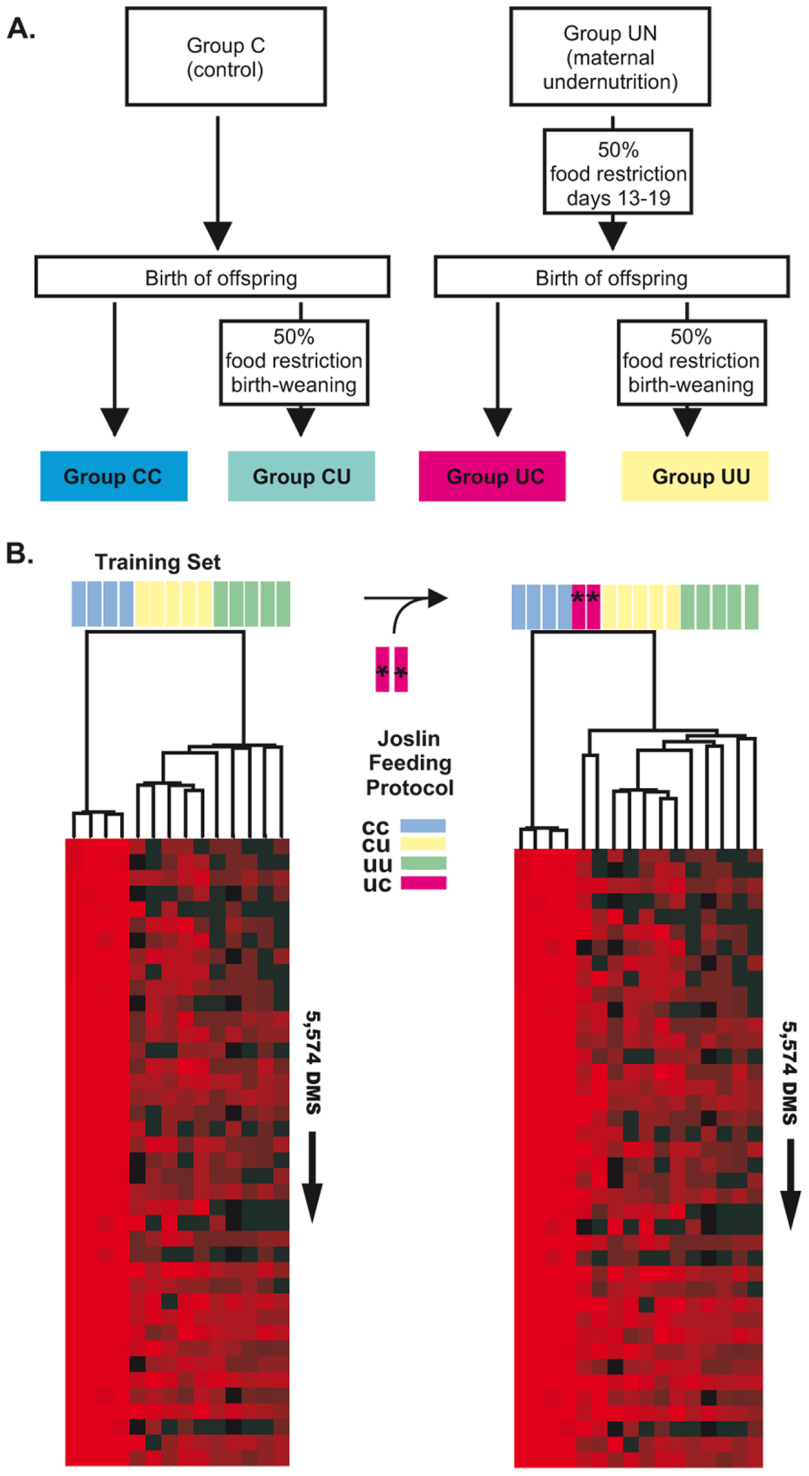
Epigenetic distances between livers of mice exposed to in utero or postnatal food restriction. The length of the branches in the hierarchical dendrogram measures the person correlation distances between digestion frequencies scored by CpGs identified as spot of *epialleles*. Shown are heat maps of the log2-transformed *digestion frequencies* (red: more than 50 reads per CpG, i.e., un-methylated; black: 0 reads per CpG, i.e., methylated). Rows represent the first 40 CpGs in the final clusters; each column represents one of the 16 tissue samples from this study. Only the field with the first 40 TS-DMS in the cluster are depicted A) Design of mouse feeding experiment (Joslin feeding protocol; adapted from [23]) B) MSCC data produced from livers of the CC, CU and UU groups. 16 outbred mice were combined with MSCC data of the 12 tissue samples. D) MSCC data produced from a liver of a C57BL/6J 12.5 d embryo was combined with MSCC data of the 12 tissue samples.

We examined to what extent these DMS can function as epigenetic signatures of each nutritional exposure. Unsupervised clustering of MSCC data obtained from 4 CC, 5 CU, and 5 UU samples regrouped experiments according to the treatment, Figure 7B. When data from a different treatment was included (UC) the samples clustered outside the previous defined groups, Figure 7B. We inferred that these DMS constitute *epialleles* that traced the nutritional history of mice during the *in utero* and/or immediate postnatal stages.

## Discussion

Based on our expanded methyl-sensitive cut counting (eMSCC) method [15], we screened for differential methylation in gDNA samples derived from liver, kidney, brain and testis. We detected 138,052 differentially methylated sites, of which 24,803 were uniquely methylated or un-methylated in one but not in the other tissues (Figure 3). Strikingly, 23,270 (93%) of these TS-DMS were hypomethylated, suggesting that un-methylation rather that methylation is the epigenetic state highlighting developmentally active loci.

In agreement with previous results obtained by Restriction Landmark Genomic Scanning (RLGS) or Methylated DNA Immunoprecipitation (MeDIP), we found that most TS-DMS represent differences between the testis and somatic tissues [17], [18], [46]–[48]. Interestingly and more often in testis, many TS-DMS co-localize in CpG islands or CpG clusters to form testis-specific un-methylated regions within gene bodies or gene deserts. The transcriptional landscape around these loci seems inconsistent with the notion that they may represent alternative promoters (Figure 1) [11], [12], [14]. A particularly interesting result is that tissue-specific differential methylation at intragenic CpG clusters correlates with tissue-specific differential expression of zinc finger protein genes (Zfp), We found 39 Zfp genes with a differentially methyled region in the 3′ terminal exon that is invariably methylated in somatic tissues and hypomethylated in testis, (Table 1). Remarkably, most of them follow a similar inverse relationship between the level of methylation at the 3′ terminal exon and gene transcriptional activity, Figure 2. It is intriguing why their TSSs remain largely un-methylated in all tissues but their intragenic CGIs seem to be preferential sites for *de novo* methylation in somatic tissues during development. The lack of differential methylation at the promoter CGIs suggests a limited role for these loci in the epigenetic transcriptional regulation, however aberrant methylation marks in the promoters of a Zfp correlates with cervical cancer [49]. Probably, the 3′ exons start the developmental program as un-methylated regions, acquiring methyl-marks as the embryo develops into adult. For instance, spatio-temporal and cell-specific methylation of these exonic-CpGs could avoid the binding of a gene repressor. Most of Zfp genes described in Table 1 belong to the C2H2 Zf family, which are enriched in KAP 1 repressor target sites. Interestingly ChiP-chip experiment detected most of the KAP1 binding sites towards the 3′ transcribed regions of these Zf-genes, [50]. Alternatively, the exonic-DNA methylation could turn the chromatin in to a Pol II elongation-permissive state. For example it has been recently shown that H3K79, H4K20, H2BK5 and H3K36m3 mark nucleosomes wrapping exonic-DNA. The tri-methylation of H3K36 is the most prominent mark and correlates positively with increased gene expression levels and stimulated transcriptional elongation [51]–[53]. In agreement with the positions of TS-DMS we found in the Zfp genes, H3K36me3 is primarily found in downstream exons, [53]. The co-occurrence of H3K36me3 and DNA hypermethylation has been recently shown in the bodies of zinc finger genes along chromosome 9, [54]. When all genes covered by this study were considered, these authors found that the co-occurrence between H3K36me3 and hypermethylation was more frequently observed at last exons or at highly expressed genes. Overall the data suggest that differential methylation of Zfp genes at 3′ exons constitutes a developmental epigenetic signature. Some of these same genes have been already identified as playing key roles in cell fate specification, [55]. It will be interesting to follow the dynamics of gene expression and methylation at these loci during embryonic development.

We observed intriguing differences in the methylation pattern of DNA derived from testis. Firstly, the largest numbers of TS-DMS were observed in testes. However, testis showed the weakest association of TS-DMS with active enhancers or tissue-specific gene expression (Tables 2, 4, and 5). Secondly, testis TS-DMS were localized at loci encoding genes involved in the embryonic development of multiple organs and anatomical structures, Figure S4 in Supporting Information S1 and Table 6. Whilst we recognize that testis includes both gametes and somatic cells, our data suggest that part of the epigenetic information involved in development would be pre-coded in the sperm genome.

The presence of differentially methylated CGIs is rarer in somatic tissues, by contrast most TS-DMS are located far from promoters, in loci with scarce CpGs and in many overlapping regions reported as tissue-specific enhancers. Indeed we found that local DNA hypomethylation highlighted active enhancers (H3K4me1 and H3K27me3) in a tissue-specific manner. Indeed, it has recently been shown that transcription factors (TF) can actively induce open chromatin spots and local de-methylation of low CpG density sequences, which ultimately constitute a footprint for DNA regulatory elements [16], [56]. Despite the scarcity of methylable sites in these elements, the methylation of critical CpGs was sufficient to prevent the TF-DNA interaction, [56].

Not only did we find active enhancers highly enriched in TS-DMS, but we also found that genes spotted with TS-DMS are expressed in a tissue-specific manner, (Tables 2 and 5) with their functional profiles matching specific functions of the corresponding tissues. Considering all our data we suggest that most of the sequences having TS-DMS must be *cis*-acting elements for tissue-specific *trans*-acting factors.

To assess the extent to which the TS-DMS could demarcate tissue-specific physiological aspects, we performed annotation enrichment analysis in the set of genes associated with these epigenetic marks. We found that the position of the TS-DMS in relation to genes defines two types of functional profiles. When TS-DMS are found in intergenic regions and distant from gene boundaries, there is a notable enrichment in processes related to embryonic development Figure S4 in Supporting Information S1.

In the instance where TS-DMS are within or very near to the borders of the gene, most of the biological processes describe physiological and organ-specific functions, (Table 6). A remarkable observation is that genes involved in specific liver functions such as, hepatic cholesterol homeostasis, response to nutrients, response to peptide hormones, plasma clearance of lipoproteins and metabolic control of xenobiotic, are distinguished in the liver genome by the presence of TS-DMS, (Figure 5 and Figure S3 in Supporting Information S1. Indeed, by ranking genes according to the number of TS-DMS and querying for enriched GO terms at the top of a ranked list, we found a selective increase in functional specificity towards lipid homeostasis, especially hepatic cholesterol management, Table 7.

We speculate that the high incidence of TS-DMS demarcating key genetic aspects of hepatic physiology could account for many of the findings that report associations between epigenetic alterations and metabolic disorders. Multiple studies have demonstrated that the nutritional state during sensitive developmental periods, including the intrauterine life and lactation periods, can “program” developmental trajectories to improve the ability of the fetus to survive in similar postnatal environments. Unfortunately, these adaptations may also modulate risk for metabolic disease in adult life. An attractive hypothesis is that these effects of nutritional exposures are mediated by altered epigenetic regulation of transcription. Whether methylation is the primary triggering event, or mediated by histone modification or effects of noncoding RNAs, remains unclear. However, our data suggest that differential methylation may mark key developmental loci, which are susceptible to nutritional or other environmental insults.

We identified thousands of sites whose differentially methylated status generated *epialleles* in the offspring of mothers subjected to different feeding protocols (Figure 7, Data Set S5). We used correlation distances to look for comparable variations in the magnitude of digestion frequencies in the sites detected as differentially methylated. Unsupervised cluster analysis shows that this set of epi-alleles characterize the nutritional history of each mouse.

In summary, in the present work we extended our previous observation that outside of CGIs and promoters there are a large number of regions with hypomethylated CpG, at low density. These regions not only outnumber the CGI but also are the loci with the greater enrichment in regulatory sequences, [15], [16]. Here we show that CpGs that are part of TS-DMS are also typically found far from promoters and outside of CGIs. TS-DMS are enriched in sequences recognized as tissue-specific enhancers and associated with tissue-specific expressed genes. Genes associated with these sites fulfill roles in the development of the corresponding tissue but also in their specific physiological functions. The identification of loci with aberrant DNA methylation marks has been largely extended during the last 5 years. Our results agree with the idea that epi-mutation rather than mutations better explain the developmental origin of diseases [57]. Most of the work on disease-related DNA methylation has focused on hypermethylation of CGI in cancer. Thus, the relevance of aberrant methylation marks outside CGI in human disease has not been well studied. Here we show that during development methylation of CpGs outside promoter or CGI seemed to be far more dynamic than commonly appreciated. We propose that the catalog of TS-DMS presented in this work, particularly those linked to the hepatic function could constitute a comprehensive epi-panel for detection and diagnosis of metabolic diseases.

## Supporting Information

Supporting Information S1 This file contains Table S1 and Figure S1–Figure S4. **Table S1**, which is a summary of the results of the short-reads-alignments to the NCBI37/mm9 assembly mouse reference genome; **Figure S1**, showing the influence of random sampling and systematic bias are canceling out during the pairwise site-by-site comparisons; **Figure S2**, Validating of the assumption of normality for the distribution of digestion frequencies (methyl sensitive cut counts) in CpGs from both lambda and mouse replicates; **Figure S3**, detailing the biological regulation branch showed in Figure 5 and **Figure S4**, showing the functional enrichment analysis results for genes proximal to TS-DMS located at intergenic regions. A description of the column headers in Data Sets S1 to S5 is provided in tables at the end of the document.
(PDF)

Data Set S1 Methyl Sensitive Cut Counting Results for CpG sites surveyed in the mouse genome
(ZIP)

Data Set S2 Methyl Sensitive Cut Counting Results for CpG sites surveyed in the lambda phage genome
(XLSX)

Data Set S3 138,052 differentially methylated sites (DMS), their genomic coordinates and their closest
(TXT)

Data Set S4 List of genes used for Gene Ontology enrichment analysis
(XLSX)

Data Set S5 List of 5,574 diet-reprogrammed differentially methylated CpGs
(XLSX)
