# Supplementary material for: A Minimal Set of Tissue-Specific Hypomethylated CpGs Constitute Epigenetic Signatures of Developmental Programming
Source: PLoS One. 2013 Sep 12;8(9):e72670. doi: 10.1371/journal.pone.0072670 (PMC3771925; doi:10.1371/journal.pone.0072670)
Supplement: Supporting Information S1 — This file contains Table S1 and Figure S1–Figure S4. Table S1, which is a summary of the results of the short-reads-alignments to the NCBI37/mm9 assembly mouse reference genome; Figure S1, showing the influence of random sampling and systematic bias are canceling out during the pairwise site-by-site comparisons; Figure S2, Validating of the assumption of normality for the distribution of digestion frequencies (methyl sensitive cut counts) in CpGs from both lambda and mouse replicates; Figure S3, detailing the biological regulation branch showed in Figure 5 and Figure S4, showing the functional enrichment analysis results for genes proximal to TS-DMS located at intergenic regions. A description of the column headers in Data Sets S1 to S5 is provided in tables at the end of the document. (PDF) [file pone.0072670.s001.pdf]

Table S1 Summary of the results of the short-reads-alignments to the NCBI37/mm9 assembly mouse reference genome

	Brain rep1	Brain rep2	Brain rep3	Kidney rep1	Kidney rep2	Kidney rep3	Liver rep1	Liver rep2	Liver rep3	Testes rep1	Testes rep2	Testes rep3
Total alignments*	40939129	40746506	53324969	48761671	51594103	56090127	49001529	40978064	53118708	45248865	45004983	55767807
Total Unique alignments**	40679379	40381793	52943713	48432180	51232896	55725845	48548295	40513942	52559486	44578879	44316500	55252505
Total multi alignments ***	3351124	3336618	4221936	4156097	4316550	4645875	4673343	4306318	5660725	6265969	5479292	5481325
Mapped to λ-genome	25419	29201	34427	27938	24135	40958	30880	26625	25409	39118	30218	35271
Mapped to mouse genome	37562586	37380687	49068606	44577636	47253418	51403294	44297306	36645121	47432574	38943778	39495473	50251211
Mouse to lambda genomes ratio	1478	1280	1425	1596	1958	1255	1434	1376	1867	996	1307	1425

The number of short sequences (reads) aligned with a one or not miss-matches; ** the number of reads aligned to unique loci; *** multi-alignments refer to reads aligning to loci that are repeated in the genome. The three biological replicates are represented by the abbreviations: rep1, rep2 and rep3.

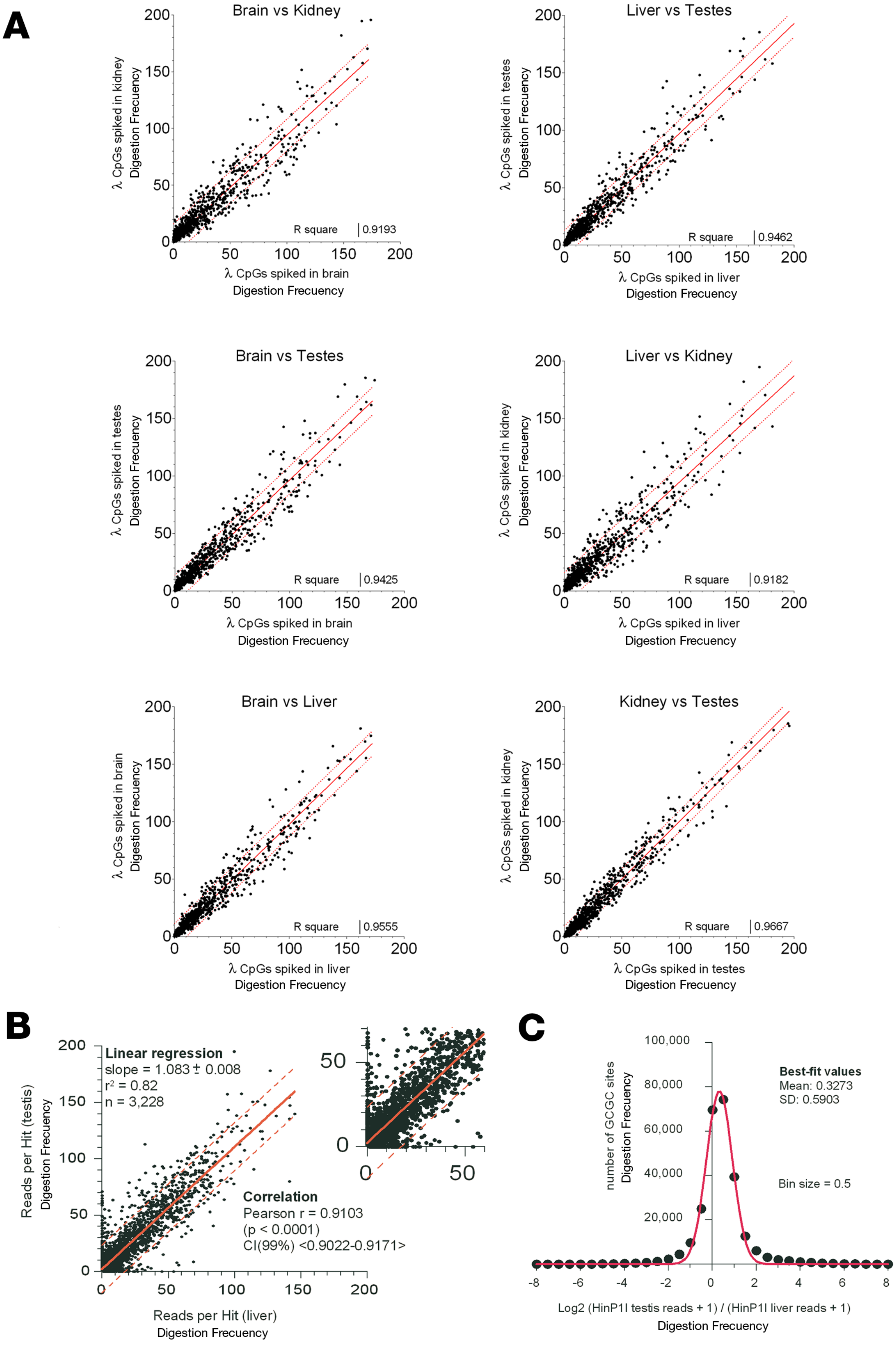
A
Brain vs Kidney
λ CpGs spiked in kidney
Digestion Frecuency
λ CpGs spiked in brain
Digestion Frecuency
R square | 0.9193
Liver vs Testes
λ CpGs spiked in testes
Digestion Frecuency
λ CpGs spiked in liver
Digestion Frecuency
R square | 0.9462
Brain vs Testes
λ CpGs spiked in testes
Digestion Frecuency
λ CpGs spiked in brain
Digestion Frecuency
R square | 0.9425
Liver vs Kidney
λ CpGs spiked in kidney
Digestion Frecuency
λ CpGs spiked in liver
Digestion Frecuency
R square | 0.9182
Brain vs Liver
λ CpGs spiked in brain
Digestion Frecuency
λ CpGs spiked in liver
Digestion Frecuency
R square | 0.9555
Kidney vs Testes
λ CpGs spiked in kidney
Digestion Frecuency
λ CpGs spiked in testes
Digestion Frecuency
R square | 0.9667
B
Linear regression
slope = 1.083 ± 0.008
r² = 0.82
n = 3,228
Correlation
Pearson r = 0.9103
(p < 0.0001)
CI(99%) <0.9022-0.9171>
Reads per Hit (testis)
Digestion Frecuency
Reads per Hit (liver)
Digestion Frecuency
C
Best-fit values
Mean: 0.3273
SD: 0.5903
Bin size = 0.5
number of GCGC sites
Digestion Frecuency
Log2 (HinP1I testis reads + 1) / (HinP1I liver reads + 1)
Digestion Frecuency

Figure S1

The influence of random sampling and systematic bias are canceling out during the pair-wise site-by-site comparisons

Differences in the level of methylation between two samples can be detected in a site-by-site multiple-comparison approach. **A)** Mouse genomic DNA samples were spiked with un-methylated foreign DNA (λ gDNA). Scatter plots represent pair-wise comparison of the number of reads aligned to the 1,202 surveyed CpG sites in the λ gDNA. In spite the measures come from equally un-methylated sites not all of them were identified with similar efficacy, the digestion frequencies at certain positions are affected by systematic biases which introduce variation in the final counts in a methylation-independent manner. However the tendency of certain CpGs to be over or underestimated are systematic and reproducible among the different experiments. **B)** Scatter plot of liver and testis digestion frequencies scored for 3,228 GCGC sites from chr5. The solid red line represents the result of a linear regression; the dashed lines defined the 95% interval of prediction. Data outside this interval could represent tissue differentially methylated CpGs. The inset shows that points located in one of the two axes represent the most marked differences. **C)** The variable "differential methylation" was defined according to:

$$\Delta\text{met} = \log_2 \frac{df^T+1}{df^L+1}$$

Where df^T and df^L represent the number of reads aligned to a particular CpG site in the testes and liver samples respectively. Despite the existence of T-DMS the distribution of methylation is expected to be highly similar for most CpG in the two tissues. Thus, when the variable Δmet is computed for several pairs of sites with similar levels of methylation the results should oscillate around zero. Figure S1-C depicts the frequency histogram of Δmet calculated for 256,394 Hinp1I sites in the liver and testes libraries. The red curve represents a single fitted Gaussian indicating that the variable Δmet follows a normal distribution with the log2 ratios scattering around zero. Differences in the library sequencing depths slightly shifted the central value from zero. Overall these results shows that the systematic error associated to the sequence environment of each CpG or the inter sites distances is similar for the identical genomes and therefore their effects are largely canceled out during the pair-wise comparisons.

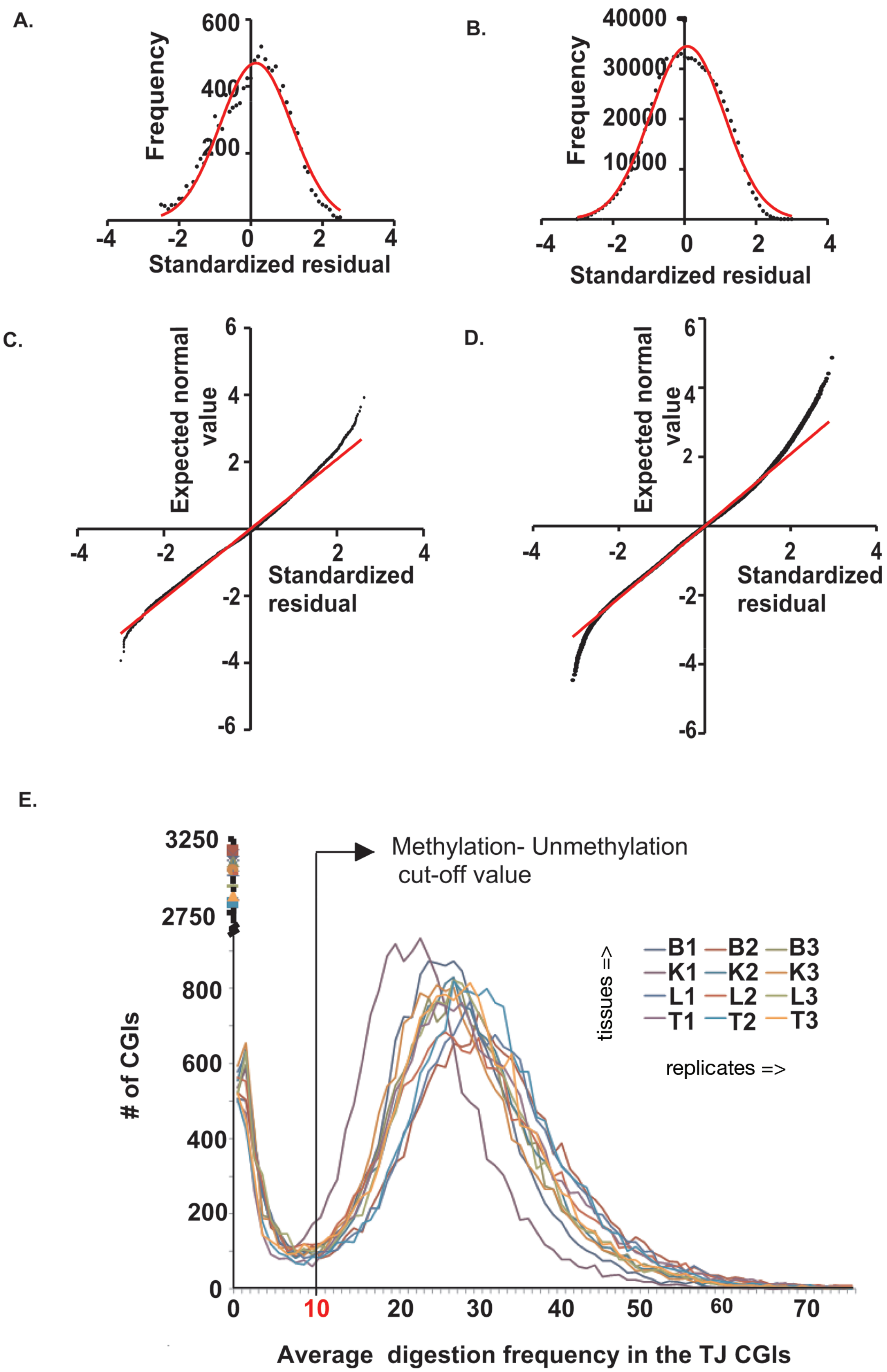
A.
Frequency
600
400
200
-4
-2
0
2
4
Standardized residual
B.
Frequency
40000
30000
20000
10000
-4
-2
0
2
4
Standardized residual
C.
Expected normal value
6
4
2
-2
-4
-6
-4
-2
2
4
Standardized residual
D.
Expected normal value
6
4
2
-2
-4
-6
-4
-2
2
4
Standardized residual
E.
3250
2750
800
600
400
200
0
# of CGIs
Methylation- Unmethylation cut-off value
tissues =>
B1 B2 B3
K1 K2 K3
L1 L2 L3
T1 T2 T3
replicates =>
0
10
20
30
40
50
60
70
Average digestion frequency in the TJ CGIs

Figure S2

Validation of the assumption of normality for the distribution of digestion frequencies (methyl sensitive cut counts) in CpGs from both lambda and mouse replicates.

Experimentally determined digestion frequencies contain deviations from an unobservable function that relates methyl sensitive counts with the level of methylation of a site, in a particular sequence environment. We studied the distribution of these deviations through replicates in a numerous set of sites both in the mouse genome as the phage lambda genome. In MSCC the standard deviation of replicates vary from one site to other, thus to compare desviations acrross multiple data points we first standardized the residual (SR) according to:

$$SR_i = \frac{df_i - \overline{df}}{sd}$$

Where, df_i represents the digestion frequency for the site i in the sample; ($\overline{df}$) is the average digestion frequency among all "i" sites included in this analysis; sd is the standard deviation associated to the previous calculated average.

These SR were depicted in a Quantile-quantile plots to compare the distribution of observed data against theoretical-expected normal distributed values.

A) Density curve of the standardized residuals in data for λ gDNA spiked in the 12-tissue gDNA samples. **B)** Density curve of 72,740 standardized residuals for CpG identification on chr1 from the tissue dataset. Each black point represents an individual CpG. The red curve is the Gaussian distribution which seems to generally fit well. **C)** and **D)** Quantile-quantile plots of lambda and mouse chromosome 1 data. The data for the most part seems to fit the Gaussian distribution, but has slightly thinner right hand tail. **E)** Takai-Jones CpG islands (TJ-CGIs) is a well documented a set of relatively large CGI that frequently lying within promoters and in most cases are fully un-methylated. Thus, the average digestion frequency calculated for CpG belonging to this CGI set is expected to represent the mode for un-methylated sites. Average digestion frequencies were calculated for each TJ-CGI. The figure shows the frequency distribution of these averages. The total number of reads before normalization in the 12 libraries were: B-1, 37,562,586; B-2, 37,380,687; B-3, 49,068,606; K-1, 44,577,636; K-2, 47,253,418; K-3, 51,403,294; L-1, 44,297,306; L-2, 36,645,121; L-3, 47,432,574; T-1, 38,943,778; T-2, 39,495,473; T-3, 50,251,211 where B, K, L and T denote brain, kidney, liver, and testes, respectively.

E

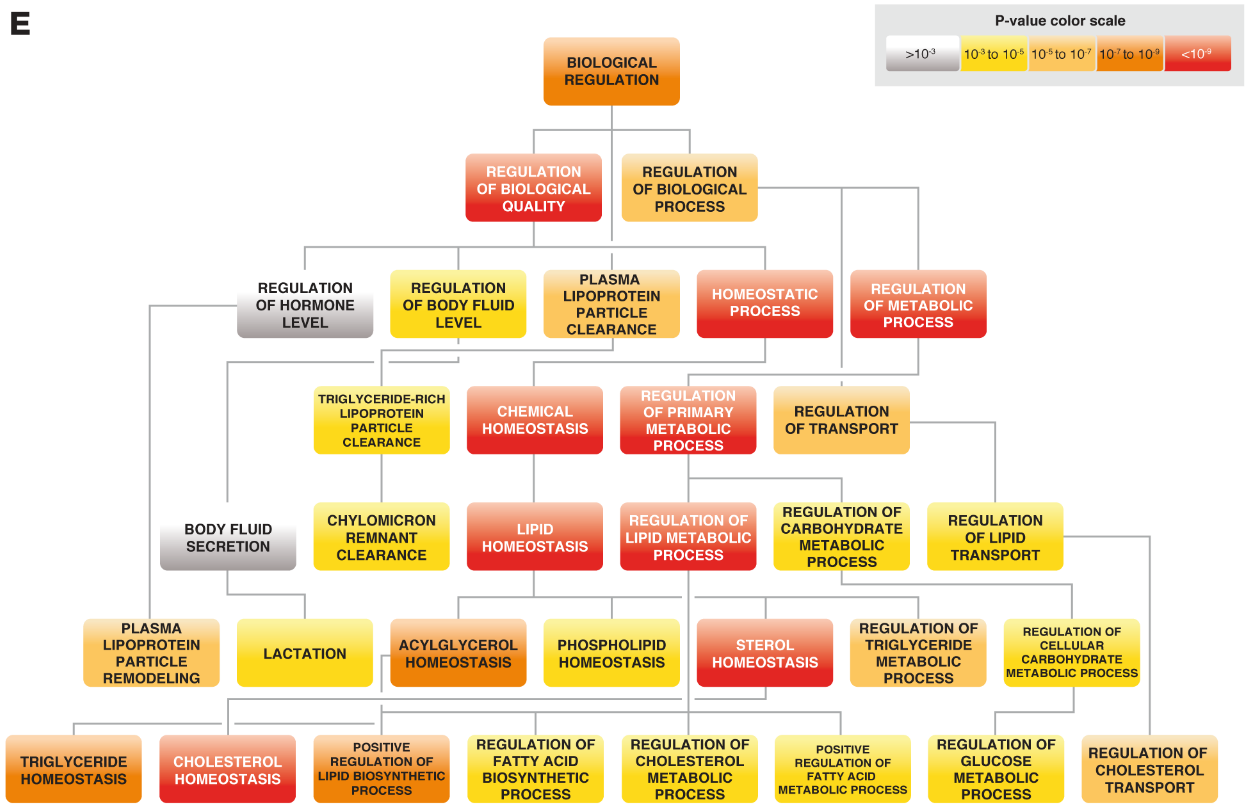

Figure S3: Detail of the biological regulation branch in Figure 5. All depicted child GO terms are significantly enriched and not redundant.

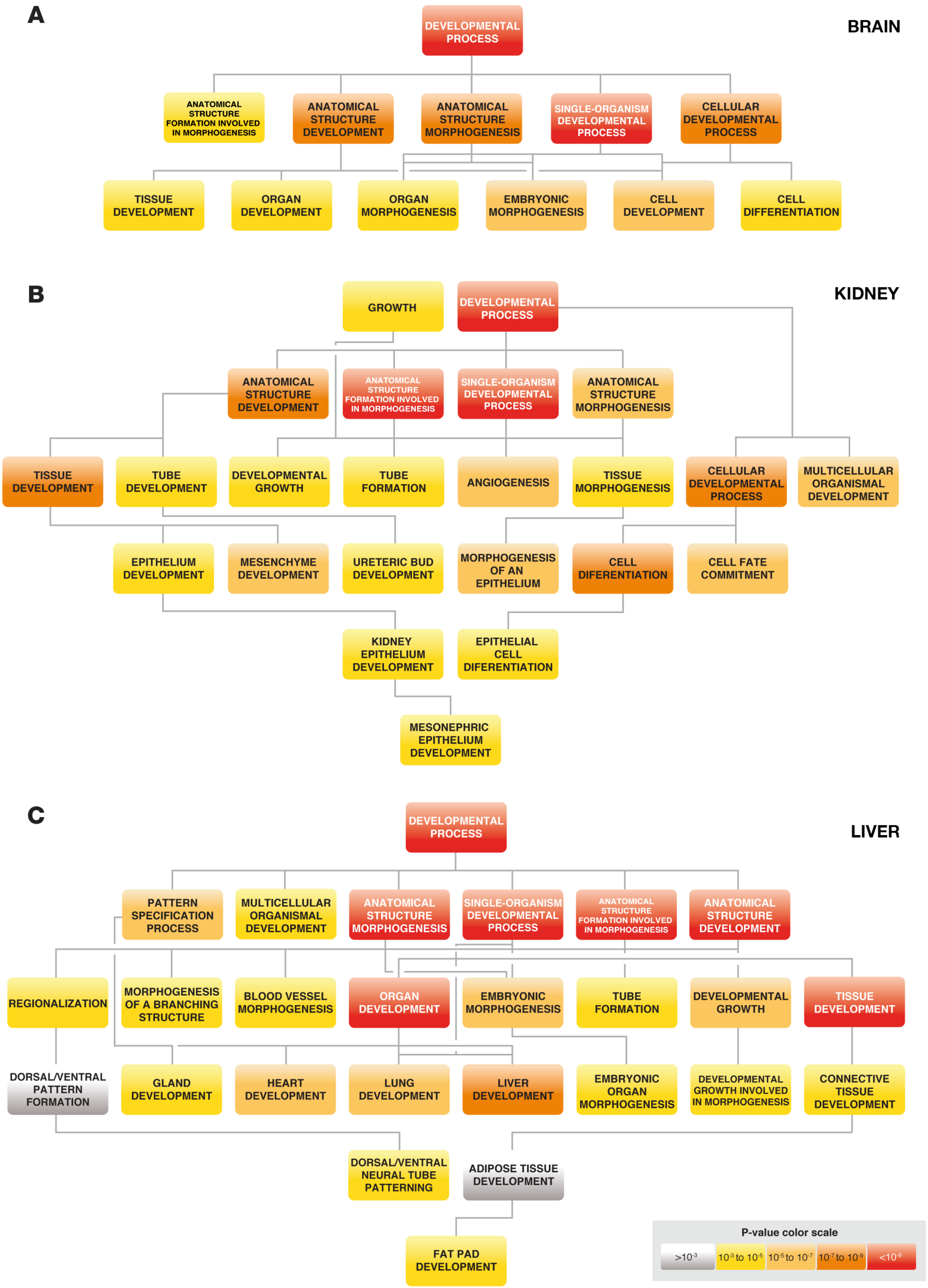
A
BRAIN
DEVELOPMENTAL PROCESS
ANATOMICAL STRUCTURE FORMATION INVOLVED IN MORPHOGENESIS
ANATOMICAL STRUCTURE DEVELOPMENT
ANATOMICAL STRUCTURE MORPHOGENESIS
SINGLE-ORGANISM DEVELOPMENTAL PROCESS
CELLULAR DEVELOPMENTAL PROCESS
TISSUE DEVELOPMENT
ORGAN DEVELOPMENT
ORGAN MORPHOGENESIS
EMBRYONIC MORPHOGENESIS
CELL DEVELOPMENT
CELL DIFFERENTIATION
B
KIDNEY
GROWTH
DEVELOPMENTAL PROCESS
ANATOMICAL STRUCTURE DEVELOPMENT
ANATOMICAL STRUCTURE FORMATION INVOLVED IN MORPHOGENESIS
SINGLE-ORGANISM DEVELOPMENTAL PROCESS
ANATOMICAL STRUCTURE MORPHOGENESIS
TISSUE DEVELOPMENT
TUBE DEVELOPMENT
DEVELOPMENTAL GROWTH
TUBE FORMATION
ANGIOGENESIS
TISSUE MORPHOGENESIS
CELLULAR DEVELOPMENTAL PROCESS
MULTICELLULAR ORGANISMAL DEVELOPMENT
EPITHELIUM DEVELOPMENT
MESENCHYME DEVELOPMENT
URETERIC BUD DEVELOPMENT
MORPHOGENESIS OF AN EPITHELIUM
CELL DIFERENTIATION
CELL FATE COMMITMENT
KIDNEY EPITHELIUM DEVELOPMENT
EPITHELIAL CELL DIFERENTIATION
MESONEPHRIC EPITHELIUM DEVELOPMENT
C
LIVER
DEVELOPMENTAL PROCESS
PATTERN SPECIFICATION PROCESS
MULTICELLULAR ORGANISMAL DEVELOPMENT
ANATOMICAL STRUCTURE MORPHOGENESIS
SINGLE-ORGANISM DEVELOPMENTAL PROCESS
ANATOMICAL STRUCTURE FORMATION INVOLVED IN MORPHOGENESIS
ANATOMICAL STRUCTURE DEVELOPMENT
REGIONALIZATION
MORPHOGENESIS OF A BRANCHING STRUCTURE
BLOOD VESSEL MORPHOGENESIS
ORGAN DEVELOPMENT
EMBRYONIC MORPHOGENESIS
TUBE FORMATION
DEVELOPMENTAL GROWTH
TISSUE DEVELOPMENT
DORSAL/VENTRAL PATTERN FORMATION
GLAND DEVELOPMENT
HEART DEVELOPMENT
LUNG DEVELOPMENT
LIVER DEVELOPMENT
EMBRYONIC ORGAN MORPHOGENESIS
DEVELOPMENTAL GROWTH INVOLVED IN MORPHOGENESIS
CONNECTIVE TISSUE DEVELOPMENT
DORSAL/VENTRAL NEURAL TUBE PATTERNING
ADIPOSE TISSUE DEVELOPMENT
FAT PAD DEVELOPMENT
P-value color scale
>10-3
10-3 to 10-5
10-5 to 10-7
10-7 to 10-9
<10-9

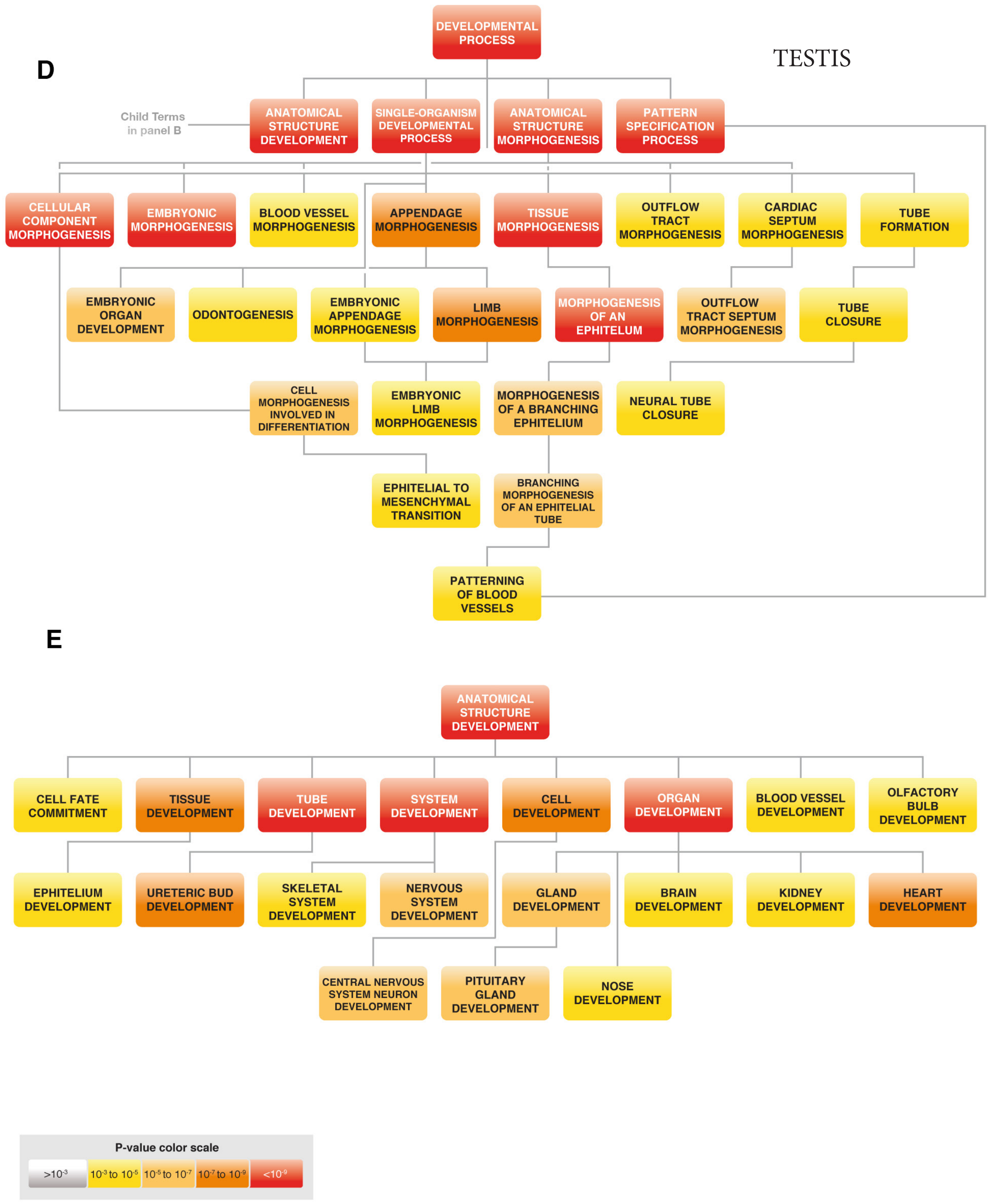

Figure S4. Functional enrichment analysis results for genes proximal to TS-DMS located at intergenic regions. These TS-DMS are located at distances bigger than 3,000 bp of any know TSS. The association between the genes used in this analysis and the above-mentioned TS-DMS has been described as “weak”, Data Set S4.

DATA SETS		
**Data Set S1: Methyl Sensitive Cut Counting Results for CpG sites surveyed in the mouse genome**		
**Column #**	**Header**	**Description**
1 or A	chrm	Identity of the chromosome containing the restriction site described in the row
2 or B	pos	Position of the chromosome containing the restriction site described in the row
3 or C	BN	Digestion frequencies scored for the restriction site described in the row. The value represents the normalized number of sequences aligned in this position of the chromosome. The results correspond to the first replicate of the brain-samples.
4 or D	BR	Same as the previous column, but second replicate of the brain-samples.
5 or E	BV	Same as the previous column, but third replicate of the brain-samples.
6 or F	KN	Digestion frequencies scored for the restriction site described in the row. The value represents the normalized number of sequences aligned in this position of the chromosome. The results correspond to the first replicate of the kidney-samples.
7 or G	KR	Same as the previous column, but second replicate of the kidney-samples.
8 or H	KV	Same as the previous column, but third replicate of the kidney-samples.
9 or I	LN	Digestion frequencies scored for the restriction site described in the row. The value represents the normalized number of sequences aligned in this position of the chromosome. The results correspond to the first replicate of the liver-samples.
10 or J	LR	Same as the previous column, but second replicate of the liver-samples.
11 or K	LV	Same as the previous column, but third replicate of the liver-samples.
12 or L	TN	Digestion frequencies scored for the restriction site described in the row. The value represents the normalized number of sequences aligned in this position of the chromosome. The results correspond to the first replicate of the testes-samples.
13 or M	TR	Same as the previous column, but second replicate of the testes-samples.
14 or N	TV	Same as the previous column, but third replicate of the testes-samples.

Data Set S2: Methyl Sensitive Cut Counting Results for CpG sites surveyed in the lambda phage genome		
**Column #**	**Header**	**Description**
1 or A	chrm	Identity of the chromosome containing the restriction site described in the row
2 or B	pos	Position of the chromosome containing the restriction site described in the row
3 or C	BN	Digestion frequencies scored for the restriction site described in the row. The value represents the normalized number of sequences aligned in this position of the chromosome. The results correspond to the first replicate of the brain-samples.
4 or D	BR	Same as the previous column, but second replicate of the brain-samples.
5 or E	BV	Same as the previous column, but third replicate of the brain-samples.
6 or F	KN	Digestion frequencies scored for the restriction site described in the row. The value represents the normalized number of sequences aligned in this position of the chromosome. The results correspond to the first replicate of the kidney-samples.
7 or G	KR	Same as the previous column, but second replicate of the kidney-samples.
8 or H	KV	Same as the previous column, but third replicate of the kidney-samples.
9 or I	LN	Digestion frequencies scored for the restriction site described in the row. The value represents the normalized number of sequences aligned in this position of the chromosome. The results correspond to the first replicate of the liver-samples.
10 or J	LR	Same as the previous column, but second replicate of the liver-samples.
11 or K	LV	Same as the previous column, but third replicate of the liver-samples.
12 or L	TN	Digestion frequencies scored for the restriction site described in the row. The value represents the normalized number of sequences aligned in this position of the chromosome. The results correspond to the first replicate of the testes-samples.
13 or M	TR	Same as the previous column, but second replicate of the testes-samples.
14 or N	TV	Same as the previous column, but third replicate of the testes-samples.

Data Set S3: 138,052 differentially methylated sites (DMS), their genomic coordinates and their closest		
**Column #**	**Header**	**Description**
1 or A	chrm	Identity of the chromosome containing the restriction site described in the row
2 or B	pos	Position of the chromosome containing the restriction site described in the row
3 or C	Tissue-specific	Indicate the tissue in which the restriction site described in the row shows tissue specificity. NA, no assigned to a particular tissue
4 or D	Distance to gene	Distance to the nearest TSS
5 or E	Region	The restriction site described in this row is mapped to specific region related of the nearest gene. See Method for more details.
6 or F	UCSC KnownGene	The gene named based on UCSC gene definition.
7 or G	Gene symbol	The official gene symbol.

Data Set S4: List of genes used for Gene Ontology enrichment analysis
The column headings are clearly self-explanatory

Data Set S5: List of 5,574 diet reprogrammed- differentially methylated CpGs		
**Column #**	**Header**	**Description**
1 or A	Chr	Identity of the chromosome containing the restriction site described in the row
2 or B	Position	Position of the chromosome containing the restriction site described in the row
3 or C	CC_ACT	Digestion frequencies scored for the restriction site described in the row. The value represents the normalized number of sequences aligned in this position of the chromosome. The results correspond to the first replicate of the brain-samples.
4 or D	CC_GAC	Same as the previous column, but second replicate of the CC-samples.
5 or E	CC_TCA	Same as the previous column, but third replicate of the CC-samples.
6 or F	CC_TGA	Same as the previous column, but forth replicate of the CC-samples.
7 or G	CU_ACT	Digestion frequencies scored for the restriction site described in the row. The value represents the normalized number of sequences aligned in this position of the chromosome. The results correspond to the first replicate of the CU-samples.
8 or H	CU_GAC	Same as the previous column, but second replicate of the CU-samples.
9 or I	CU_REG	Same as the previous column, but third replicate of the CU-samples.
10 or J	CU_TCA	Same as the previous column, but forth replicate of the CU-samples.
11 or K	CU_TGA	Same as the previous column, but fifth replicate of the CU-samples.
12 or L	UC_TCA	Digestion frequencies scored for the restriction site described in the row. The value represents the normalized number of sequences aligned in this position of the chromosome. The results correspond to the first replicate of the UC-samples.
13 or M	UC_TGA	Same as the previous column, but second replicate of the UC-samples.

14 or N	UU_ACT	Digestion frequencies scored for the restriction site described in the row. The value represents the normalized number of sequences aligned in this position of the chromosome. The results correspond to the first replicate of the UU-samples.
15 or O	UU_GAC	Same as the previous column, but second replicate of the UU-samples.
16 or P	UU_REG	Same as the previous column, but third replicate of the UU-samples.
17 or Q	UU_TCA	Same as the previous column, but forth replicate of the UU-samples.
18 or R	UU_TGA	Same as the previous column, but fifth replicate of the UU-samples.
19 or S	GeneRegion	The restriction site described in this row is mapped to specific region related of the nearest gene. See Method for more details.
20 or T	Gene-UCSC	The gene named based on UCSC gene definition.
21 or U	Name	The official gene symbol.
22 or V	RefSeq	The gene named based on RefSeq definition.
23 or W	Details	Description of the gene.
